# Supplementary material for: The KRAB Zinc Finger Protein Roma/Zfp157 Is a Critical Regulator of Cell-Cycle Progression and Genomic Stability
Source: Cell Rep. 2016 Apr 14;15(4):724–34. doi: 10.1016/j.celrep.2016.03.078 (PMC4850358; doi:10.1016/j.celrep.2016.03.078)
Supplement: Document S1. Supplemental Experimental Procedures and Figures S1–S4 [file mmc1.pdf]

Cell Reports, Volume *15*

Supplemental Information

The KRAB Zinc Finger Protein Roma/Zfp157 Is a Critical Regulator of Cell-Cycle Progression and Genomic Stability

Teresa L.F. Ho, Guillaume Guilbaud, J. Julian Blow, Julian E. Sale, and Christine J. Watson

Figure S1 (related to Figure 1)

A

5dG						
	**WT1**	**WT2**	**WT3**	***Roma***$^{-/-}$**1**	***Roma***$^{-/-}$**2**	***Roma***$^{-/-}$**3**
**AVG%**	21.69	25.46	24.94	46.01	45.96	47.31
**SD**	8.35	8.73	9.16	6.45	5.79	6.02
p = 9.52E-04						
**10dL**						
	**WT1**	**WT2**	**WT3**	***Roma***$^{-/-}$**1**	***Roma***$^{-/-}$**2**	***Roma***$^{-/-}$**3**
**AVG%**	4.04	4.95	4.78	29.02	27.8	29.5
**SD**	6.58	5.43	6.95	12.48	9.14	10.46
p = 2.49E-05						

B

Small Intestine						
	**WT1**	**WT2**	**WT3**	***Roma***$^{-/-}$**1**	***Roma***$^{-/-}$**2**	***Roma***$^{-/-}$**3**
**AVG%**	29.35	29.92	30.10	45.47	44.58	44.97
**SD**	1.65	0.34	0.57	1.13	0.62	0.30
p = 1.84E-06						
**Spleen**						
	**WT1**	**WT2**	**WT3**	***Roma***$^{-/-}$**1**	***Roma***$^{-/-}$**2**	***Roma***$^{-/-}$**3**
**AVG%**	2.25	1.73	1.78	8.98	9.27	8.89
**SD**	0.49	0.19	0.18	0.41	0.80	0.43
p = 1.19E-05						
**Thymus**						
	**WT1**	**WT2**	**WT3**	***Roma***$^{-/-}$**1**	***Roma***$^{-/-}$**2**	***Roma***$^{-/-}$**3**
**AVG%**	3.39	3.16	3.36	20.11	20.01	19.75
**SD**	0.29	0.39	0.25	0.57	0.13	0.15
p = 1.65E-07						

C

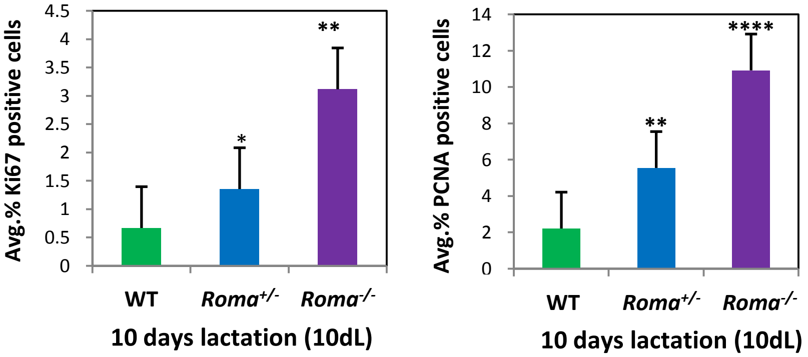

D

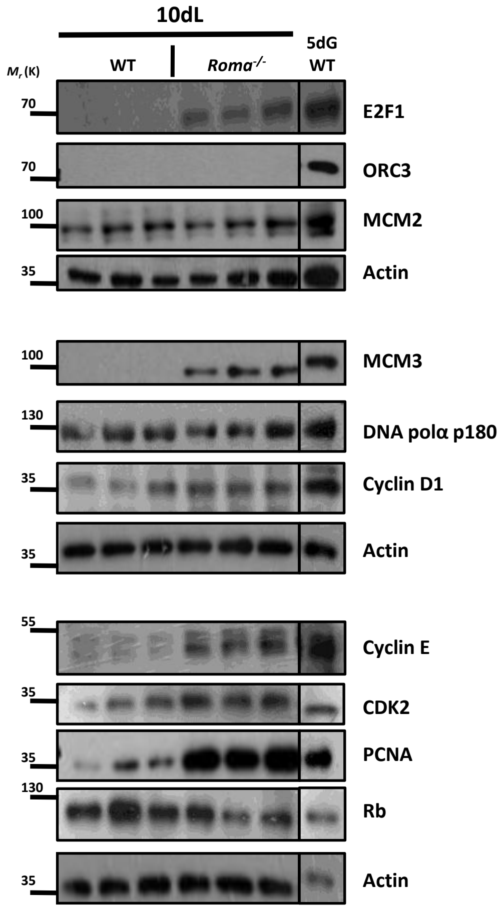

Figure S2 (Related to Figure 2)

A

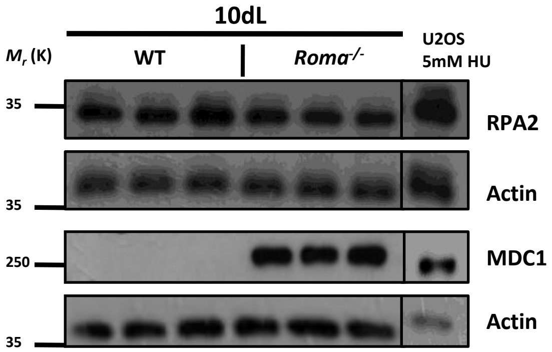

B

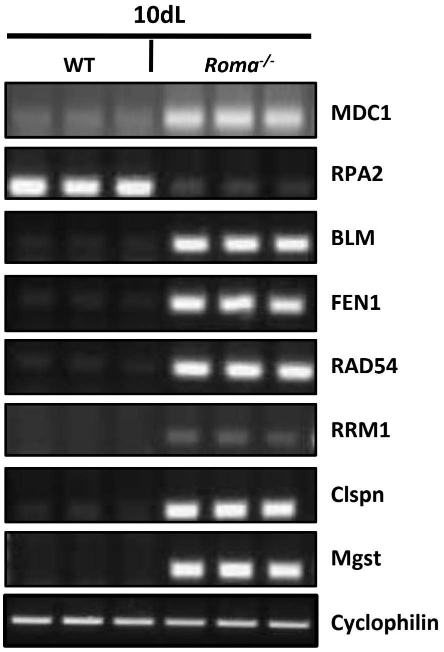

C

WT	Chr 1	Chr 2	Chr 3
Breaks		1	
Fragile site	2		2
Deletions			
Translocations			
Duplication			

Roma-/-	Chr1	Chr2	Chr4	Chr5	Chr6	Chr7	Chr8	Chr9	Chr10	Chr11	Chr12	Chr14	Chr15	Chr16	Chr17	Chr18	ChrX
Breaks	6	5	3	9	6	2	3	3		4	2	1	2	7	3	2	2
Fragile site	5	3	7	3	3	2		2	10	2				3		1	2
Deletions	4	4	8	6	2	2	2	1		2	2	4	2	3		3	1
Translocations		2&14	4&12<br>4&18		6&2<br>6&17	7&12<br>7&17		9&18		11&12<br>11&13<br>11&15		14&3<br>14&15<br>14&17		16&19	17&3<br>17&8<br>17&9<br>17&19	18&12	X&3<br>X&10<br>X&13
Duplication	2		3	3		2		2		1	1	2					

Roma-/-	Chr1	Chr2	Chr5	Chr6	Chr7	Chr8	Chr10	Chr11	Chr12	Chr13	Chr14	Chr15	Chr16	Chr17	Chr18	Chr19	ChrX
Premature separation of sister chromatids (freq.)	1	4	2	2	2	4	15	10	1	5	4	6	1	5	4	1	3

Figure S3 (Related to Figure 3)

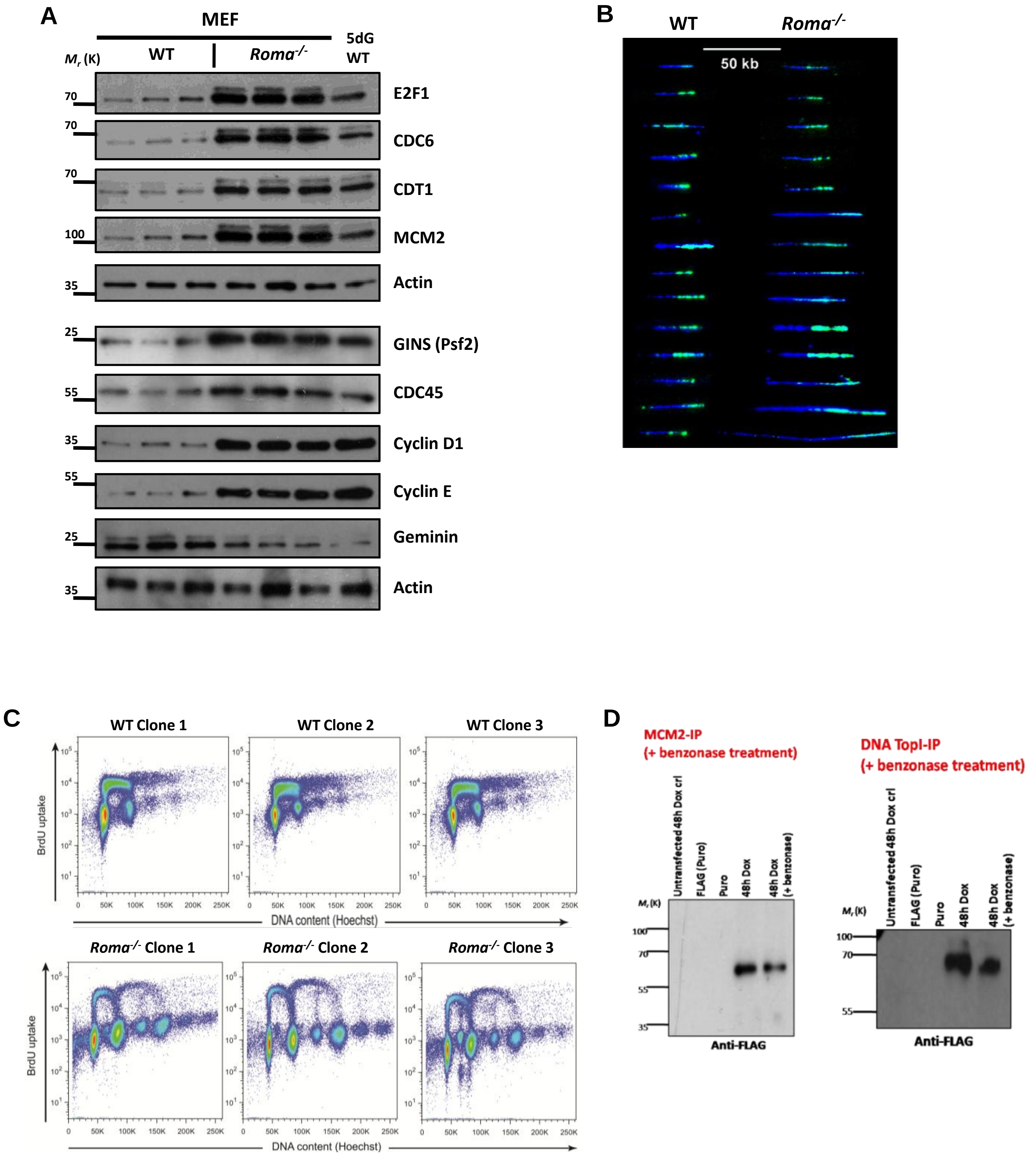

Figure S4 (Related to Figure 4)

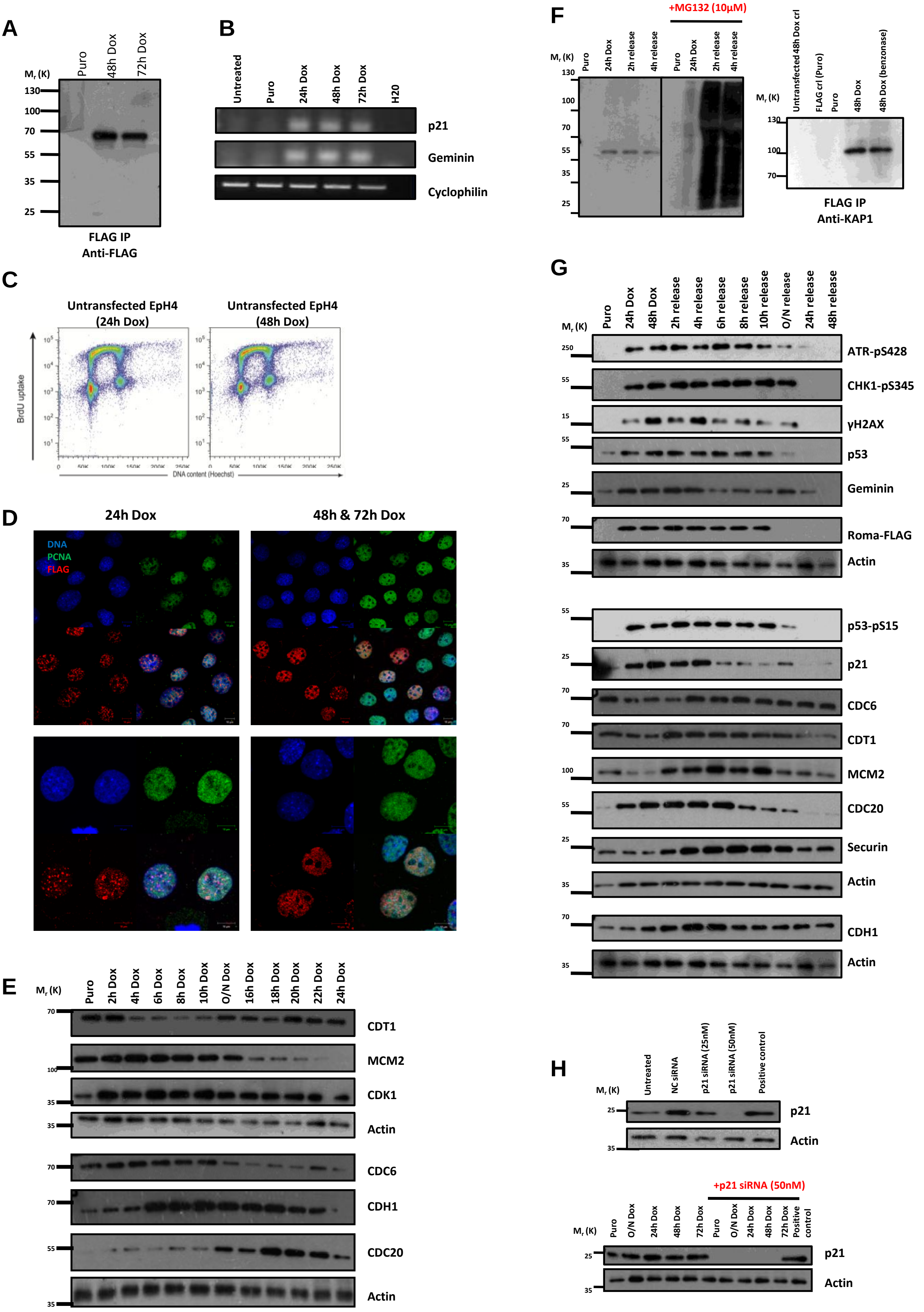

Supplemental Figure Legends

Figure S1 (Related to Figure 1) (A) Average percentages of EdU+ cells per alveolar structure in WT and *Roma*$^{-/-}$ mammary glands at 5dG and 10dL (n=3 mice per group, 5dG: p = 9.52E-04, 10dL: p = 2.49E-05, Student's t-test). **(B)** *In vivo* IP injections of EdU in WT and *Roma*$^{-/-}$ mice at 7-8 weeks of age and various organs harvested. Number of EdU+ cells per field of view were counted at 20X magnification for small intestine and 40X magnification for spleen and thymus and presented as an average percentage of positive cells to total cells counted. (n=3 mice per group, Small intestine: p = 1.84E-06, Spleen: p = 1.19E-05, Thymus: p = 1.65E-07, Student's t-test). **(C)** Immunofluorescence analysis of WT, *Roma*$^{+/-}$ and *Roma*$^{-/-}$ mammary glands at 10dL with Ki67 and PCNA. Average percentages of Ki67+ or PCNA+ cells per field of view at 20X magnification were calculated. Data is presented as mean and error bars represent standard deviation (SD). (n=3 mice per group, Ki67: WT vs *Roma*$^{+/-}$: p = 0.024; WT vs *Roma*$^{-/-}$: p = 0.008; PCNA: WT vs *Roma*$^{+/-}$: p = 0.001; WT vs *Roma*$^{-/-}$: p = 4.73E-06; Student's t-test). **(D)** Protein extracts were prepared from WT and *Roma*$^{-/-}$ mammary glands at 10dL (n=3 mice per group) followed by immunoblot analysis of cell cycle factors. Extract from 5dG WT gland used as proliferation control.

Figure S2 (Related to Figure 2) (A) Immunoblot analysis of cell cycle checkpoints and DNA damage response markers in WT and *Roma*$^{-/-}$ mammary glands at 10dL (n=3 mice per group). Extract from U2OS cells treated with 5 mM HU was used as a damage control**. (B)** PCR analysis of various cell cycle checkpoints and DNA damage response markers in WT and *Roma*$^{-/-}$ mammary glands at 10dL (n=3 mice per group) were conducted. Cyclophilin used as an internal control. **(C)** Summary of gross chromosomal rearrangements in WT and *Roma*$^{-/-}$ mammary epithelial cells isolated from mammary glands after a full natural wean (n=3 mice per group).

Figure S3 (Related to Figure 3) (A) Immunoblot analysis of cell cycle factors in WT and *Roma*$^{-/-}$ MEFs (n=3 clones per group). Extract from 5dG WT gland was used as a proliferation control. **(B)** A selection of representative fibers analysed. **(C)** Primary WT and *Roma*$^{-/-}$ MEFs at passage 5 were pulsed with BrdU followed by cell cycle analysis using flow cytometry (n=3 clones per group). **(D)** Protein extracts were prepared from EpH4 cells under the following conditions: untransfected EpH4 cells+48h doxycycline, EpH4+control FLAG vector (Puromycin selection), EpH4+Roma-FLAG vector (Puromycin selection, no induction), EpH4+Roma-FLAG vector + 48h doxycycline (Puromycin selection, induction), EpH4+Roma-FLAG vector+48h doxycycline+benzonase treatment (Puromycin selection, induction) followed by Mcm2 and DNA Topoisomerase I immunoprecipitation respectively and immunoblot against Roma-FLAG showing association.

Figure S4 (Related to Figure 4) (A) Protein extracts were prepared from EpH4 cells under puromycin selection (without doxycycline induction) and at various timepoints after doxycycline-induced Roma-FLAG expression. FLAG immunoprecipitation was conducted followed by immunoblot analysis of Roma-FLAG expression. **(B)** RNA was extracted from EpH4 cells under puromycin selection (without doxycycline induction) and at various timepoints after doxycycline-induced Roma-FLAG expression and PCR analysis of p21^{Cip1} and geminin was conducted. Cyclophilin used as an internal control. **(C)** Untransfected EpH4 cells were treated with doxycyline for 48h before pulsing with BrdU followed by cell cycle analysis. **(D)** Representative immunofluorescence analysis of PCNA and Roma-FLAG in EpH4 cells at various timepoints after doxycycline-induced Roma-FLAG expression. Scale bars = 10μm. **(E)** Immunoblot analysis of cell cycle factors in EpH4 cells during a timecourse of doxycycline-induced Roma-FLAG

expression was conducted**.** **(F)** Protein was extracted from EpH4 cells over a timecourse of release from doxycycline-induced Roma-FLAG expression (+/- MG132) followed by anti-FLAG immunoblot analysis. Protein extracts were prepared from EpH4 cells under the stated conditions followed by FLAG immunoprecipitation and immunoblot against KAP1 showing association with Roma-FLAG. **(G)** Immunoblot analysis of cycle checkpoint, DNA damage response and cell cycle factors in EpH4 cells during a timecourse of release from doxycycline-induced Roma-FLAG expression was conducted**. (H)** Protein was extracted from EpH4 cells under the following treatment conditions – untreated, scrambled siRNA, 25nM p21^{Cip1} siRNA, 50nM p21^{Cip1} siRNA followed by immunoblot for p21^{Cip1}. Extract from 5dG WT gland was used as a proliferation control. Protein was extracted from EpH4 cells under the stated treatment conditions (+/- 50nM p21^{Cip1} siRNA) followed by immunoblot against p21^{Cip1}.

Video S1 (Related to Figure 3)

Video clip of WT MEFs during live cell imaging.

Video S2 (Related to Figure 3)

Video clip of *Roma*$^{-/-}$ MEFs during live cell imaging.

Table S1 (Related to Figure 3)

A separate Excel Workbook containing raw data from DNA fiber analysis.

Supplemental Experimental Procedures

Quantification of EdU-positive cells in other tissues

For intestine, images were taken at 20X magnification and 10 fields of view were counted. For spleen and thymus, images were taken at 40X magnification and 5 fields of view were counted. Analysis was conducted as stated in Experimental Procedures.

Quantification of PCNA and Ki67-positive MECs

For the analysis of PCNA+ and Ki67+ cells, images were taken at 20X magnification and 10 fields of view were analyzed per gland. Number of PCNA+ or Ki67+ cells were expressed as a percentage of total number of cells. Statistical significance was assessed using unpaired two-tailed Student's *t*-tests in Microsoft Excel (TTEST).

PCR

Other primers used include (PCR parameters can be found under Experimental Procedures):

Cyclin B1 – forward (CAGAGTTCTGAACTTCAGCCTG); reverse (TTGTGAGGCCACAGTTCACCAT)

CDK1 – forward (ACAGAGAGGGTCCGTCGTAA); reverse (ATTGCAGTACTGGGCACTCC)

MDM2 – forward (TTAGTGGCTGTAAGTCAGCAAGA); reverse (CCTTCAGATCACTCCCACCT)

RPA2 – forward (TGTTGGCGGCAGAATCATGG; reverse (CTCGTCAAGTGGCTCCATCAA)

BLM – forward (AAGCCTGAGTGAGGATCATGG); reverse (TGACAGACACATCACCCTCTG)

FEN1 – forward (GCTAGCTGCTTAAGGCTCGT); reverse (AGGAGCAATGGCTTCTTCCTAC)

RAD54 – forward (TGGACCCAAGCCTCATCCTC)

reverse (TAAGCTCCTCCTCATCCTGGC)

RRM1 – forward (ACGAAGCACCCTGACTATGC); reverse (TGGCAGAATTCAGGCGATCC)

Clspn – forward (GCACTGCAGAAGAATGCCAG); reverse (TTCCTTGAGTTTCGGGGAGC)

Mgst – forward (AAGATTGGAAGCATGGCCGA); reverse (CTGGGTTGGCAAAAACCTTGT)
