# Supplementary figures and images for: The KRAB Zinc Finger Protein Roma/Zfp157 Is a Critical Regulator of Cell-Cycle Progression and Genomic Stability
Source: Cell Rep. 2016 Apr 14;15(4):724–34. doi: 10.1016/j.celrep.2016.03.078 (PMC4850358; doi:10.1016/j.celrep.2016.03.078)

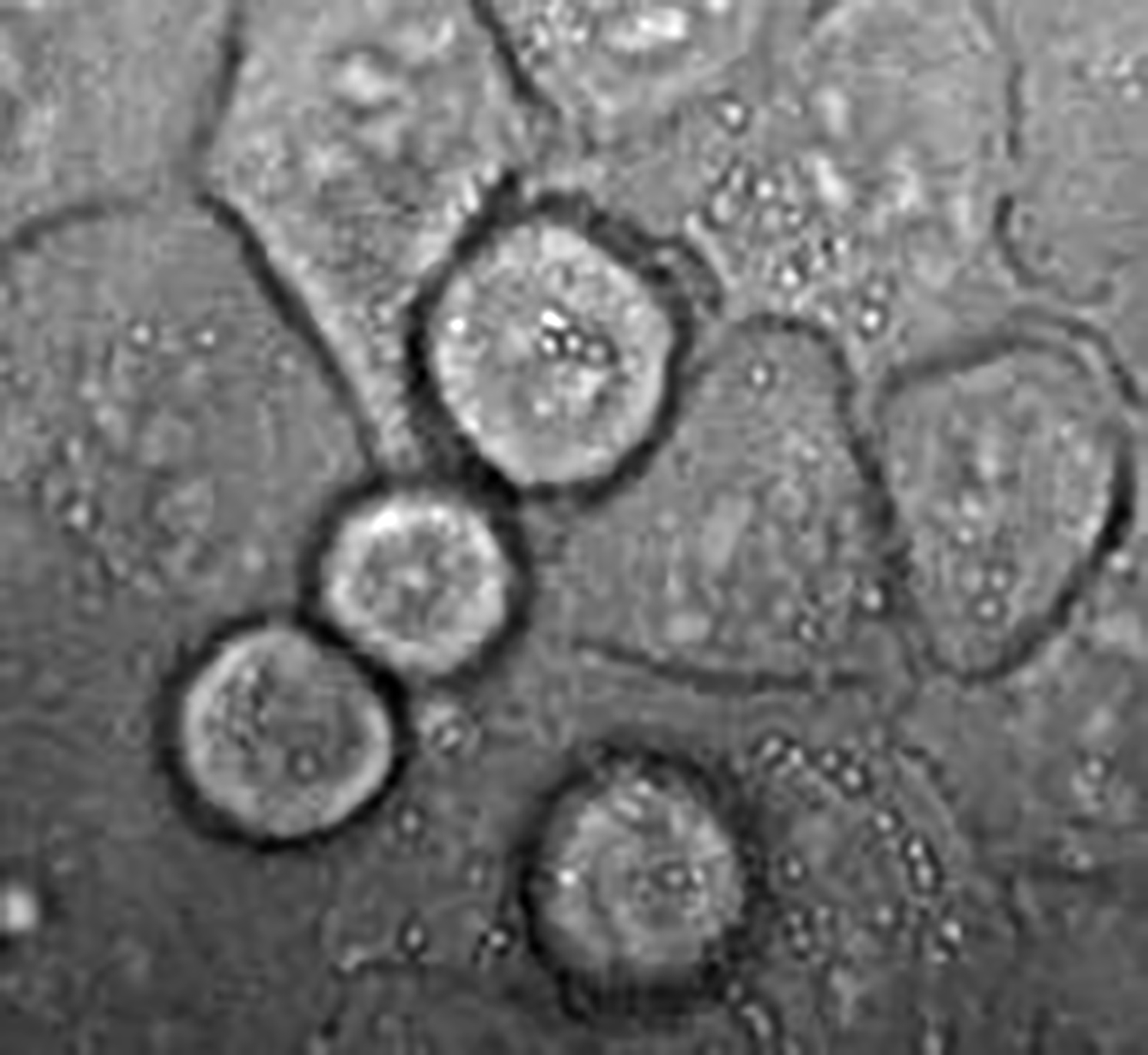

Supplement: Movie S1. Video Clip of WT MEFs during Live-Cell Imaging, Related to Figure 3 [file mmc3.jpg]

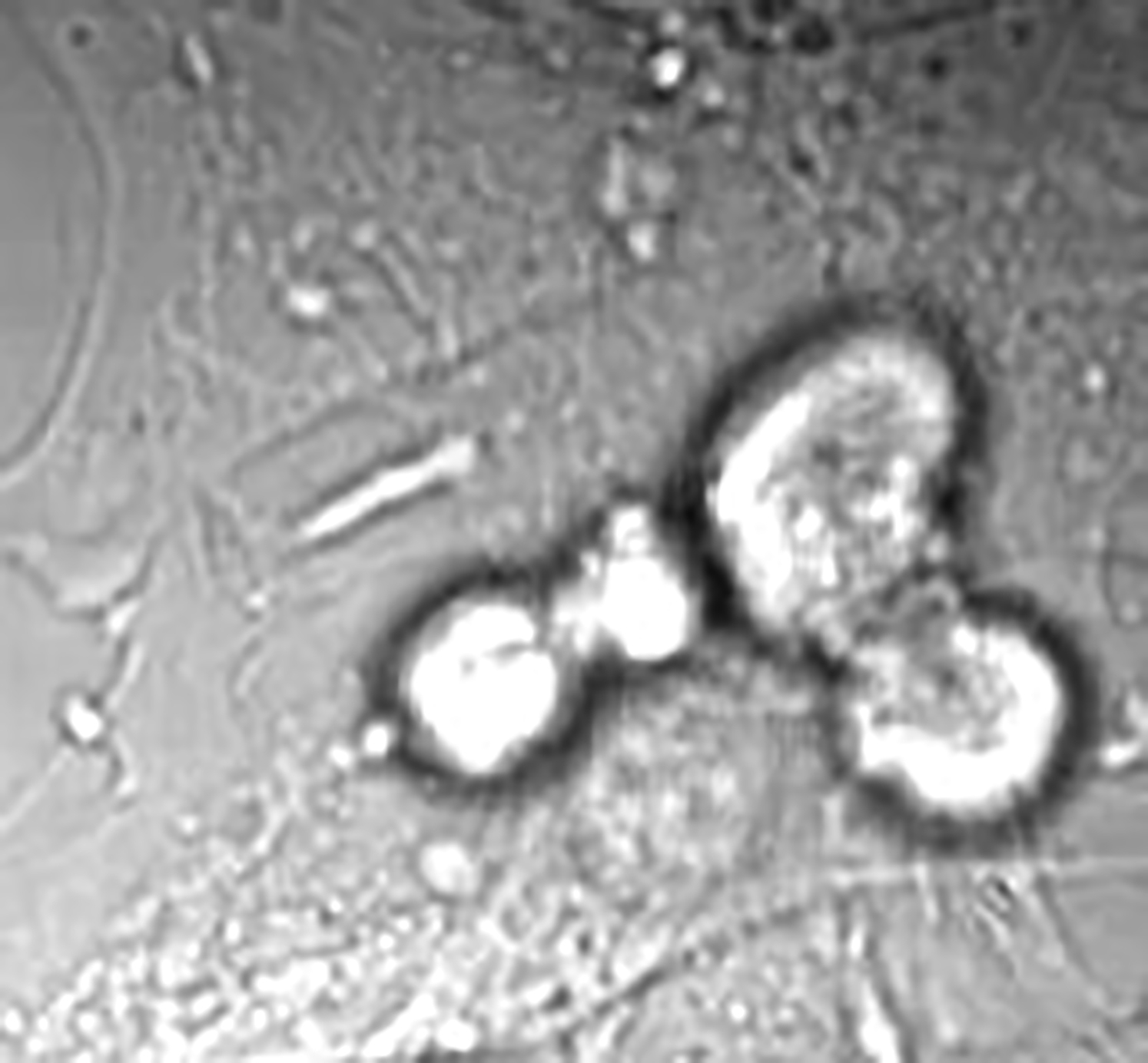

Supplement: Movie S2. Video Clip of Roma–/– MEFs during Live-Cell Imaging, Related to Figure 3 [file mmc4.jpg]
