# Supplementary material for: The KRAB Zinc Finger Protein Roma/Zfp157 Is a Critical Regulator of Cell-Cycle Progression and Genomic Stability
Source: Cell Rep. 2016 Apr 14;15(4):724–34. doi: 10.1016/j.celrep.2016.03.078 (PMC4850358; doi:10.1016/j.celrep.2016.03.078)
Supplement: Document S2. Article plus Supplemental Information [file mmc5.pdf]

Cell Reports

Report

# The KRAB Zinc Finger Protein Roma/Zfp157 Is a Critical Regulator of Cell-Cycle Progression and Genomic Stability

## Graphical Abstract

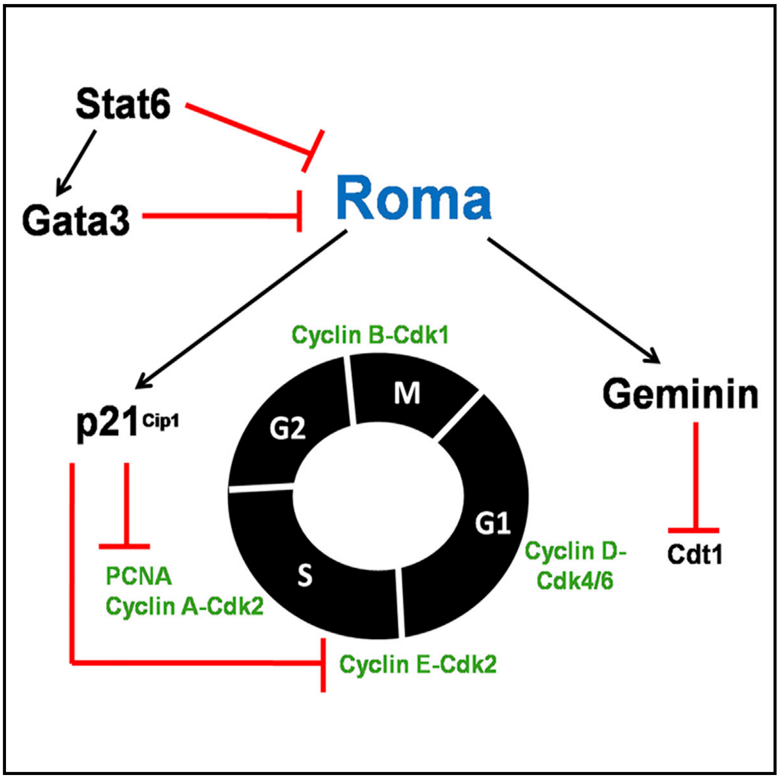


## Authors

Teresa L.F. Ho, Guillaume Guilbaud, J. Julian Blow, Julian E. Sale, Christine J. Watson

## Correspondence

cjw53@cam.ac.uk

## In Brief

Ho et al. demonstrate that the transcriptional regulator Roma, a member of the KRAB/Zinc finger protein family, controls cell-cycle progression and replication fork velocity. Loss of Roma in lactating mammary gland results in replication stress and activation of DNA damage signaling pathways.

## Highlights

- The KRAB-domain zinc finger protein Roma/Zfp157 regulates proliferation
- Roma regulates expression of the cell-cycle regulators p21$^{Cip1}$ and geminin
- Roma regulates replication fork dynamics and genomic stability
- Ablation of Roma in mouse embryonic fibroblasts leads to endoreduplication

Ho et al., 2016, Cell Reports *15*, 724–734
April 26, 2016 ©2016 The Authors
http://dx.doi.org/10.1016/j.celrep.2016.03.078

OPEN ACCESS
CellPress

Cell Reports
Report

# The KRAB Zinc Finger Protein Roma/Zfp157 Is a Critical Regulator of Cell-Cycle Progression and Genomic Stability

Teresa L.F. Ho,[1] Guillaume Guilbaud,[2] J. Julian Blow,[3] Julian E. Sale,[2] and Christine J. Watson[1,*]
[1]Department of Pathology, University of Cambridge, Tennis Court Road, Cambridge CB2 1QP, UK
[2]MRC Laboratory of Molecular Biology, Francis Crick Avenue, Cambridge CB2 0QH, UK,
[3]Centre for Gene Regulation and Expression, College of Life Sciences, University of Dundee, Dow Street, Dundee DD1 5EH, UK
*Correspondence: cjw53@cam.ac.uk
http://dx.doi.org/10.1016/j.celrep.2016.03.078

## SUMMARY

**Regulation of DNA replication and cell division is essential for tissue growth and maintenance of genomic integrity and is particularly important in tissues that undergo continuous regeneration such as mammary glands. We have previously shown that disruption of the KRAB-domain zinc finger protein Roma/Zfp157 results in hyperproliferation of mammary epithelial cells (MECs) during pregnancy. Here, we delineate the mechanism by which Roma engenders this phenotype. Ablation of Roma in MECs leads to unscheduled proliferation, replication stress, DNA damage, and genomic instability. Furthermore, mouse embryonic fibroblasts (MEFs) depleted for Roma exhibit downregulation of p21$^{Cip1}$ and geminin and have accelerated replication fork velocities, which is accompanied by a high rate of mitotic errors and polyploidy. In contrast, overexpression of Roma in MECs halts cell-cycle progression, whereas siRNA-mediated p21$^{Cip1}$ knockdown ameliorates, in part, this phenotype. Thus, Roma is an essential regulator of the cell cycle and is required to maintain genomic stability.**

## INTRODUCTION

The adult mammary gland undergoes cycles of proliferation, differentiation, and regression with every pregnancy. Mammary epithelial progenitor cells initially undergo rapid proliferation during pregnancy before differentiation into specialized milk-producing alveolar cells during lactation. (Watson and Khaled, 2008). We have shown previously that proliferation of alveolar cells during pregnancy is reduced when the transcription factor Stat6 is ablated (Khaled et al., 2007). Microarray analysis identified the Krüppel-associated box (KRAB) zinc finger protein, Zfp157 (herein called Roma—regulator of mammary alveologenesis), as the most highly upregulated gene in *Stat6*$^{-/-}$ mammary glands at day 5 gestation (5dG). KRAB-Zfps constitute the largest family of transcriptional regulators, are found only in tetrapods, and are generally transcriptional repressors (Urrutia, 2003). Various functions for these DNA binding proteins are just beginning to be elucidated (Lupo et al., 2013).

Generation of a Roma-LacZ reporter/functional knockout (hereafter named *Roma*$^{-/-}$) mouse revealed, as anticipated, accelerated alveologenesis in pregnant females in concert with elevated levels of proliferation (Oliver et al., 2012). Unexpectedly, the ratio of ERα/PR/Gata3-expressing cells to pStat5 cells was dramatically skewed in favor of the latter when Roma was ablated. Both Stat5 and Gata3 are essential transcription factors for pregnancy-induced development and ablation of Gata3 from luminal epithelium during gestation results in lactation failure arising from death of epithelial cells (Asselin-Labat et al., 2007; Kouros-Mehr et al., 2006). Surprisingly, this lactation failure was rescued by coincident loss of Roma. Thus, we concluded that Roma is a master regulator of the alveolar lineage and that Gata3 is superfluous when Roma is not expressed (Oliver et al., 2012). However, the role of Roma in cell-cycle progression has not been investigated.

The cell cycle is exquisitely regulated by formation of unique cyclin and cyclin-dependent kinase (CDK) complexes that regulate entry into and drive progression of, different phases of the cell cycle. CDK function is tightly regulated by p21$^{Cip1}$ and downregulation or loss of p21$^{Cip1}$ is often associated with cell-cycle dysregulation and aberrant proliferation (Coqueret, 2003). Furthermore, p21$^{Cip1}$ is a critical enforcer of the G1 and G2 checkpoints (Shaltiel et al., 2015) and is a component of p53-mediated responses, and its downregulation compromises the cellular response to stress. Although much effort has been expended to determine mechanisms of p21$^{Cip1}$ regulation, our understanding of p21$^{Cip1}$ regulation in the cell cycle is incomplete.

The accurate and precise duplication of DNA occurs during S phase and to ensure that the genome is duplicated only once; coordinated licensing and initiation of replication origins is critical (Blow and Hodgson, 2002). Geminin is a major inhibitor of replication licensing that prevents excessive firing and unscheduled re-firing of origins (Klotz-Noack et al., 2012). Furthermore, modulation of replication fork velocity is important to circumvent failure to duplicate genomic regions harboring natural barriers, and p21$^{Cip1}$ has been implicated in assisting replication at such regions (Hosfield et al., 1998). Other factors that regulate fork progression include the Cdc45-GINS-Mcm2–7


This is an open access article under the CC BY license (http://creativecommons.org/licenses/by/4.0/).
CrossMark

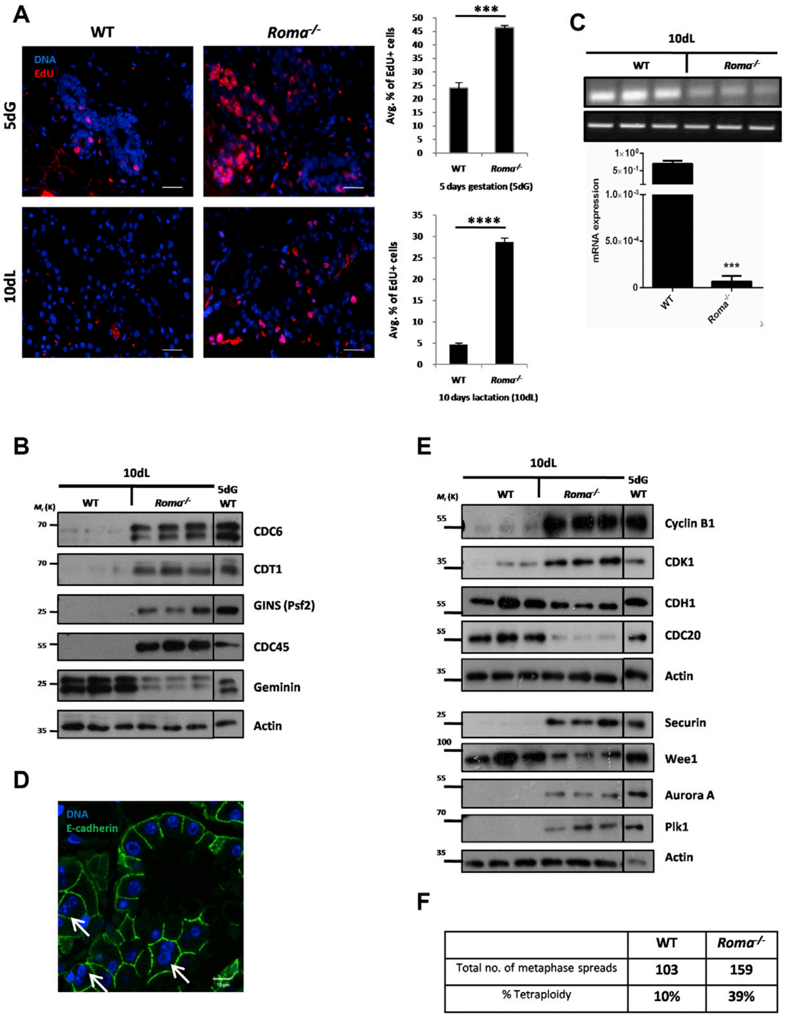


| | WT | Roma-/- |
|---|---|---|
| Total no. of metaphase spreads | 103 | 159 |
| % Tetraploidy | 10% | 39% |

**Figure 1. Absence of *Roma* Leads to Uncontrolled Proliferation**

(A) Immunofluorescence analysis of EdU incorporation in mammary gland tissue sections from WT and *Roma*$^{-/-}$ mice at 5dG and 10dL, 24 hr following injection in vivo. Scale bars, 10 μm. The number of EdU$^{+}$ and EdU$^{-}$ cells was counted and presented as an average percentage. Data are presented as the mean, and error bars represent SD (5dG: p = 9.52E−04, 10dL: p = 2.49E−05, Student's t test).

(B) Protein extracts were prepared from WT and *Roma*$^{-/-}$ glands at 10dL followed by immunoblot analysis of cell-cycle factors as indicated. Protein extract from 5dG WT gland was used as a proliferation control.

(C) PCR and quantitative real-time PCR analysis of geminin in extracts from 10dL WT and *Roma*$^{-/-}$ glands (p = 0.0002, Student's t test).

(D) Immunofluorescence analysis of E-cadherin staining (green) with binucleate cells indicated (white arrows). Nuclei are stained with DAPI (blue). Scale bars, 10 μm.

(E) Immunoblot analysis of cell-cycle factors in extracts of WT and *Roma*$^{-/-}$ glands at 10dL. Protein extract from 5dG WT gland was used as a proliferation control.

(F) Primary MECs were isolated from WT and *Roma*$^{-/-}$ glands at full wean, and metaphase spreads were prepared. Karyotype analysis was carried out, and tetraploidy was determined by counting the number of chromosomes.

See also Figure S1.

(CMG) helicase and DNA topoisomerases. These unwind DNA, resolve topological tension, and maintain DNA polymerase processivity in concert with proliferating cell nuclear antigen (PCNA). Dysregulation of these events results in re-replication, replication stress, aneuploidy, and genomic instability, significant events in tumorigenesis and tumor heterogeneity (Burrell et al., 2013). Here, we show that Roma is a critical component of the mammalian cell cycle that controls replication dynamics and cell-cycle progression.

## RESULTS

### Ablation of Roma Leads to Uncontrolled Proliferation and Prevents Developmentally Programmed Cell-Cycle Exit

We have previously shown that ablation of Roma results in hyperproliferation of MECs in early pregnancy. This is not due to changes in systemic hormones, and, although the rate of ductal elongation is accelerated in pubertal *Roma*$^{-/-}$ mice, there are no obvious defects in adult glands (data not shown). The increase in proliferation in *Roma*$^{-/-}$ glands at 5dG was further supported by an increased proportion of 5-ethynyl-2′-deoxyuridine (EdU) positive mammary epithelial cells (MECs) (Figures 1A and S1A). Notably, at 10 days lactation (10dL) when cells are terminally differentiated (Faraldo et al., 2002) and non-proliferative in control glands (Figures 1A and S1A), proliferation is still evident in *Roma*$^{-/-}$ glands, suggesting that Roma is required for the transition from cell-cycle progression to quiescence. Increased EdU labeling was evident also in intestine, spleen, and thymus of young *Roma*$^{-/-}$ mice (Figure S1B).

Immunoblot analysis of 10dL mammary tissue extracts revealed an increase in levels of replication licensing proteins (Cdc6, Cdt1, and Mcm3) and replisome components (Cdc45 and GINS) (Figures 1B and S1D), while the licensing inhibitor geminin was strikingly downregulated at protein and RNA levels (Figures 1B and 1C). This pattern is consistent with failure to downregulate replication licensing for cell-cycle exit (Blow and Hodgson, 2002). Furthermore, *Roma*$^{-/-}$ MECs at 10dG were found to be undergoing aberrant re-replication (data not shown), a process suppressed by geminin (Melixetian et al., 2004). Immunofluorescence analysis revealed a significant increase in cells expressing Ki67 and PCNA in *Roma*$^{-/-}$ 10dL glands (Figure S1C). Interestingly, we observed more binucleated cells in *Roma*$^{-/-}$ in comparison to wild-type (WT) glands (Figure 1D), indicating cytokinesis failure. The relative increase in Cdh1 levels compared to Cdc20 in *Roma*$^{-/-}$ MECs (Figure 1E) could

contribute to mitotic slippage (Floyd et al., 2008). This possibility is supported by elevated levels of securin and the mitotic kinases Aurora A and Plk1 (Figure 1E) that could impede sister chromatid separation (Petronczki et al., 2008). To investigate further, we performed karyotype analysis on cells from WT and *Roma*$^{-/-}$ glands after a full lactation and natural wean and found that Roma deficiency correlated with an approximately 4-fold increase in tetraploidy (Figure 1F). This suggests that the unscheduled proliferation during lactation leads to cell-cycle dysregulation, with chromosomal missegregation and instability.

### Unscheduled Proliferation in the Absence of *Roma* Leads to Replication Stress and Activation of the DNA Damage Response

Replication stress results in phosphorylation of the ssDNA binding protein RPA2 on residue T21 by the ATR/Chk1 kinases. We noted RPA2 (pT21) foci by immunofluorescence analysis of 10dL *Roma*$^{-/-}$ glands (Figure 2A). Furthermore, collapse of stalled forks to form double-strand breaks (DSBs) is evidenced by γH2AX foci and presence of large 53BP1 foci (Figure 2A), reminiscent of 53BP1-OPT domains observed in G1 cells (Harrigan et al., 2011) that mark replication stress-mediated DNA lesions arising from the previous S phase (Lukas et al., 2011). Immunoblot analysis revealed activation of ATR-Chk1-driven S and G2 phase checkpoints and p53 activation as evidenced by p53 (pS15) levels and upregulation of Gadd45 (Figures 2B and S2A). Intriguingly, another major downstream target of p53, p21$^{Cip1}$, which is an important effector of cell-cycle arrest upon checkpoint activation, is not correspondingly upregulated in the absence of *Roma* (Figure 2B). Indeed, quantitative real-time PCR analysis indicated that p21$^{Cip1}$ was transcriptionally downregulated in *Roma*$^{-/-}$ glands compared to WT (Figure 2C). RNA levels of other DNA damage responders such as Blm, Fen1, and Rrm1, which localize to stalled forks are also upregulated (Figure S2B).

These data indicate that Roma insufficiency results in unscheduled proliferation, replication stress, and DNA damage, which would activate the G2/M checkpoint. A key player is Wee1, which negatively regulates CDK1 to prevent mitosis (Lianga et al., 2013), and Wee1 levels are diminished in *Roma*$^{-/-}$ mammary glands (Figure 1E). The increased proportion of binucleated cells upon *Roma* loss (Figure 1D) further supports the notion of defects in G2/M checkpoint arrest and/or checkpoint bypass probably arising from the decreased levels of Wee1 and p21$^{Cip1}$.

Hence, it is not surprising that we observed an increase in gross structural chromosomal rearrangements in *Roma*$^{-/-}$ glands compared to WT (Figures 2D and S2C). Such genomic damage could arise from DNA replication errors and/or S phase checkpoint defects (Myung et al., 2001). Since key genes such as p21$^{Cip1}$ and geminin are transcriptionally dysregulated in the absence of *Roma*, these effects could be mediated initially at the transcriptional level via recruitment of Roma and additional co-factors, such as KAP-1, to the promoters of a subset of these genes.

### Roma Regulates Replication Fork Dynamics

These data highlight a role for Roma in cell-cycle regulation. To facilitate mechanistic analysis, we derived primary mouse embryonic fibroblasts (MEFs) from WT and *Roma*$^{-/-}$ mid-gestation embryos. Immunoblot analysis of a panel of cell-cycle regulators recapitulated the observations in 10dL mammary glands (Figure S3A), providing us with a tractable in vitro system. We investigated replication dynamics by pulse-labeling MEFs with iododeoxyuridine (IdU) followed by chlorodeoxyuridine (CldU) after which DNA fibers were spread and analyzed (Figure 3A). Surprisingly, *Roma*$^{-/-}$ MEFs exhibited an approximately 45% increase in overall replication fork velocity compared to WT (1.6 ± 0.4 and 1.1 ± 0.02 kb/min, respectively) (Figures 3A and S3B). Furthermore, in *Roma*$^{-/-}$ MEFs, inter-origin distances were larger than in WT (130 ± 7 and 105 ± 4 kb, respectively) (Figure 3B). *Roma*$^{-/-}$ MEFs are characterized by, on average, one less replication origin per megabase than WT MEFs (4.6/Mb and 6.1/Mb, respectively) (Table S1), which could be a consequence of the faster moving forks in *Roma*$^{-/-}$ cells inactivating adjacent replication origins (Blow and Ge, 2008).

The increased replication fork speeds could result from failure to pause at DNA secondary structures and repetitive DNA sequences (Mazouzi et al., 2014) and could lead, potentially, to under-replicated areas, replication stress, and DNA damage response activation. Supporting this possibility, immunoblot analysis showed ATR/Chk1 and p53 activation (Figure 3C). These profiles are remarkably similar to those observed in mammary gland suggesting that aberrant DNA replication occurs also in the absence of *Roma* in vivo. Again, levels of p21$^{Cip1}$ were not correspondingly upregulated to reinforce the G2/M checkpoint.

2D cell-cycle analysis of WT and *Roma*$^{-/-}$ MEFs by pulse labeling with bromodeoxyuridine (BrdU) to identify cells in S phase revealed striking aneuploidy and polyploidy in *Roma*$^{-/-}$ MEFs after only five passages (Figures 3D and S3C). Measurement of the proportion of cells in either S phase (high BrdU content) or in G1/G2/M (low BrdU content) demonstrated that strikingly fewer *Roma*$^{-/-}$ MEFs are in S phase (7%) compared to WT (35%). *Roma*$^{-/-}$ MEFs also incorporated BrdU at a higher rate, consistent with them progressing faster through S phase.

During live-cell imaging of *Roma*$^{-/-}$ MEFs, we noted that, while they are able to form the mitotic cleavage furrow characteristic of telophase, they subsequently fail to divide (Movies S1 and S2). This impairment of cytokinesis in vitro correlates with elevated levels of Plk1, Aurora A, and securin (Figure 3E), that would impede sister chromatid separation. SAC components such as BubR1 are elevated in *Roma*$^{-/-}$ MEFs (Figure 3E) as are levels of Mps1, which contributes to inhibition of proper mitotic exit in the presence of misaligned chromosomes (Althoff et al., 2012). Taken together, these observations suggest aberrant mitotic bypass and ploidy.

One possible interpretation of the increased fork velocities observed in Roma$^{-/-}$ MEFs is that, in the absence of *Roma*, downregulation of p21$^{Cip1}$ stabilizes interactions between PCNA and DNA polymerase δ (Sexton et al., 1997). p21$^{Cip1}$ has a role also in facilitating fork pausing at natural barriers through interactions with PCNA and Fen1 (Hosfield et al., 1998). Immunoprecipitation experiments in EpH4 normal MECs expressing a doxycycline-inducible Roma FLAG-tagged construct (Figure 3F) suggest that Roma interacts with Mcm2, Mcm3, and DNA Topoisomerase I although the binding domains required for these interactions have yet to be determined (Figure S3D). We propose

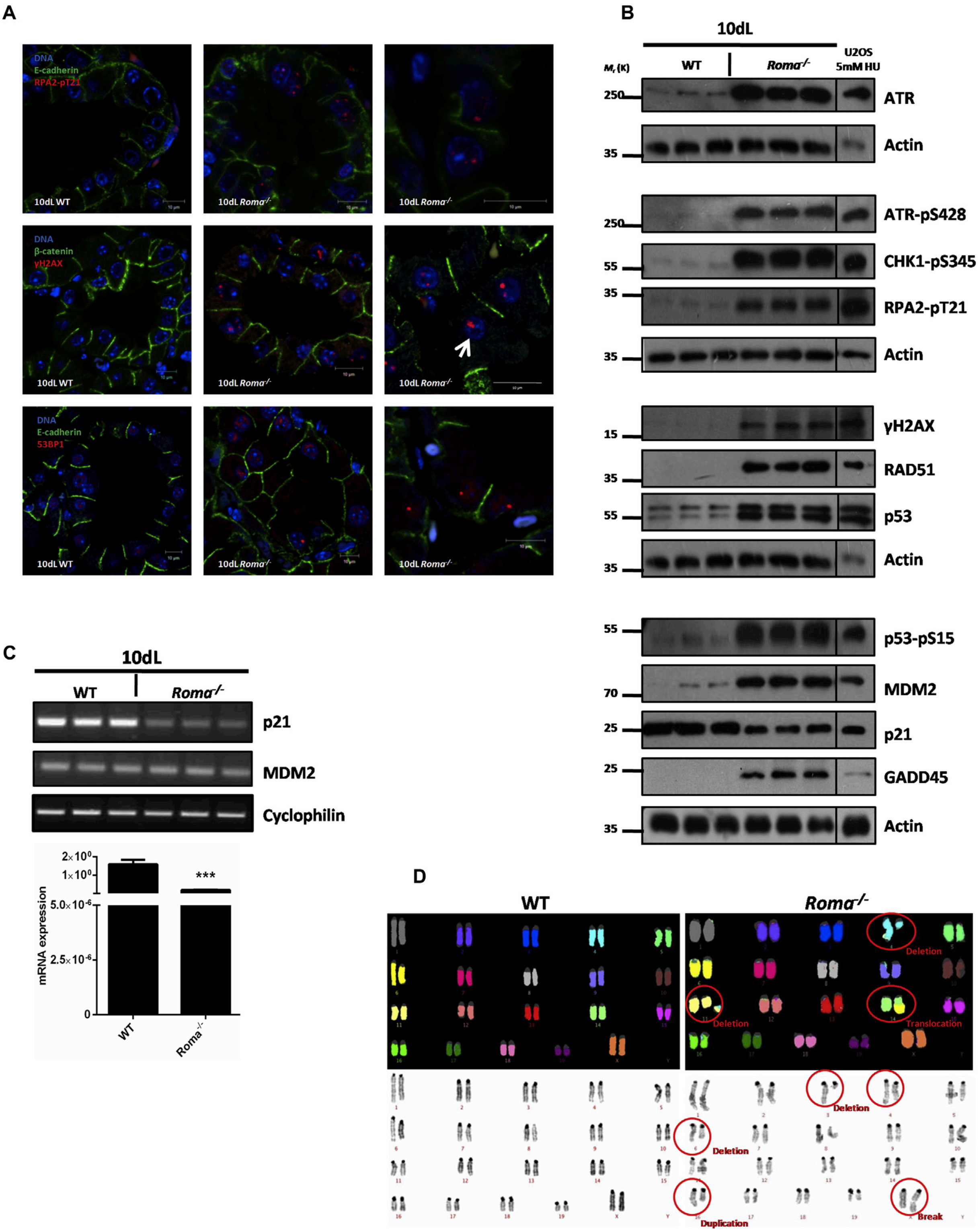


**Figure 2. Absence of *Roma* Leads to Activation of the DNA Damage Response and Genomic Instability**

(A) Representative immunofluorescence analysis of RPA2-pT21, γH2AX, and 53BP1 foci in tissue sections from WT and *Roma*$^{-/-}$ glands at 10dL. The white arrow indicates nucleolar γH2AX. Scale bars, 10 μm. The higher magnification panels on the right have been chosen to highlight the foci and are not zoomed images of the left panels.

*(legend continued on next page)*

that loss of association between Roma and these key replication factors in *Roma*$^{-/-}$ MEFs could potentially influence their recruitment and function at replication forks, contributing to an increase in replication fork speeds. This could lead to under-replication of some regions of the genome with coincident DNA damage that would activate the G2/M checkpoint enforced by p21$^{Cip1}$. However, given the downregulation of p21$^{Cip1}$ when Roma is ablated (Figures 3C and 3E), we posit that cells are able to bypass the checkpoint and enter mitosis. Furthermore, it has been shown that p21$^{Cip1}$ is critical in preventing S phase entry after aberrant mitotic exit through cyclin E-CDK2 inhibition (Stewart et al., 1999). Hence, the downregulation of p21$^{Cip1}$ would explain both the polyploidy and the continued cell-cycle progression in cells lacking Roma.

Another possible interpretation of the above data is that *Roma*$^{-/-}$ cells are undergoing endoreduplication without appropriate cell division. The re-replication or endoreduplication of cells arrested in G2 is strongly enhanced by the loss of p21$^{Cip1}$ (Khan and Wahl, 1998) or geminin (Klotz-Noack et al., 2012).

## Overexpression of Roma Leads to Cell-Cycle Collapse

Next, we sought to investigate the impact of Roma overexpression in EpH4 normal MECs using a doxycycline-inducible Roma FLAG-tagged construct (Figures S4A and S4C). Roma overexpression inhibited proliferation as evidenced by a decrease in total cell numbers over a 6-day time course (Figure 4A). Cell morphology was altered, with cells switching from a cuboidal epithelial form to a more elongated, spindle-shaped phenotype after prolonged induction of Roma expression (Figure 4B). Immunofluorescence analysis with an anti-FLAG antibody revealed an aggregation of Roma-FLAG into distinct foci after 24 hr doxycycline induction (Figure S4D). These foci are reminiscent of replication foci (Toledo et al., 2013), supporting the involvement of Roma at the replication fork level.

BrdU analysis of EpH4 cells overexpressing Roma revealed that high levels of Roma led to S phase collapse with a lack of DNA synthesis (Figure 4C, top panel). It is striking that high levels of Roma do not lead to arrest at a specific cell-cycle phase but rather, cells are halted in G1, S, and G2/M. The sub-G1 population also suggests that Roma may induce cell death. Immunoblot analysis revealed a rapid increase in p21$^{Cip1}$ levels within 6 hr that was independent of p53 activation (Figures 4D and S4E). Transcriptional upregulation of p21$^{Cip1}$ occurred within 4 hr of Roma expression being induced (Figure 4E, top panel). p21$^{Cip1}$ can block origin firing by inhibiting CDK activity and binding PCNA, precluding its interaction with DNA polymerase $\delta$ (Waga et al., 1994). p21$^{Cip1}$ inhibition of G2, M, and G1 CDKs would further contribute to the full cell-cycle arrest observed. Another factor that was rapidly upregulated in response to Roma overexpression was geminin. Protein levels are increased after 8-hr induction (Figure 4D), while an increase in RNA was evident by 6 hr (Figure 4E, top panel). Increased geminin would arrest cells in late mitosis and G1 through Cdt1 inhibition and promotion of Cdt1 degradation by the E3 ubiquitin ligases SCF$^{Skp2}$ and Cul4-Ddb1$^{Cdt2}$, leading to the rapid decrease in Cdt1 levels observed (Figure S4E).

Upon release from doxycycline, Roma levels decline rapidly, possibly by ubiquitin-mediated degradation (Figure S4F), and cells previously arrested in S phase appear to resume replication within 2 hr of release (Figure 4C, bottom panel). p21$^{Cip1}$ RNA and protein levels start to decrease by 4 and 6 hr, respectively (Figure 4E, bottom panel; Figure S4G), independent of p53. Notably, arrested cells that re-enter the cell cycle appear to undergo endoreduplication after 6 hr (Figure 4C, bottom panel). Cells released from p21$^{Cip1}$-induced G2 arrest undergo endoreduplication as p21$^{Cip1}$ interferes with checkpoints that prevent re-entry into S phase without prior mitosis (Niculescu et al., 1998). The cell death observed after 8 hr is likely the fate of endoreduplicating cells upon G1 checkpoint activation (Figure 4C, bottom panel).

Together, these results suggest that Roma overexpression rapidly, and reversibly, leads to S phase collapse and cell-cycle arrest. Given that p21$^{Cip1}$ is transcriptionally upregulated when Roma is overexpressed, we knocked down p21$^{Cip1}$ (Figure S4H) and observed that this alleviated the cell-cycle arrest induced by Roma overexpression, suggesting that excess Roma can mediate S phase arrest primarily through upregulation of p21$^{Cip1}$ (Figure 4F). However, since p21$^{Cip1}$ knockdown did not completely prevent cell-cycle arrest but rather led to an accumulation of cells in G1, we suggest that geminin, which is also transcriptionally regulated by Roma (data not shown) could be responsible for blocking S phase entry by inhibiting replication licensing (Figures 4D and 4E). Clearly, levels of Roma need to be exquisitely regulated in order to maintain cell-cycle progression with excess, or insufficient, Roma resulting in cell-cycle arrest or unscheduled DNA replication and genomic instability.

## DISCUSSION

KRAB-domain zinc finger proteins (KRAB-Zfps) comprise a large family of rapidly evolving DNA binding proteins that are unique to tetrapods and impart tissue specific functions (Urrutia, 2003). Roma is a KRAB-Zfp with a previously unsuspected role as a regulator of mammalian cell-cycle progression through modulation of multiple cell-cycle components and control of replication fork velocity. We have identified p21$^{Cip1}$ and geminin as critical downstream targets of Roma and demonstrated physical interaction between Roma and components of the replication machinery such as Mcm2/3 and DNA Topoisomerase I. Loss of Roma results in increased replication fork speeds and

(B) Immunoblot analysis of cell-cycle checkpoint and DNA damage response in extracts from WT and *Roma*$^{-/-}$ glands at 10dL. Extracts from U2OS cells treated with 5mM HU were used as a damage control.
(C) PCR analysis of p21$^{Cip1}$ and MDM2 and quantitative real-time PCR analysis of p21$^{Cip1}$ in extracts from 10dL WT and *Roma*$^{-/-}$ glands (p = 0.0007, Student's t test).
(D) Representative spectral karyotypes (top) and metaphase spreads (bottom panel) prepared from MECs isolated from WT and *Roma*$^{-/-}$ glands after a full natural wean.
See also Figure S2.

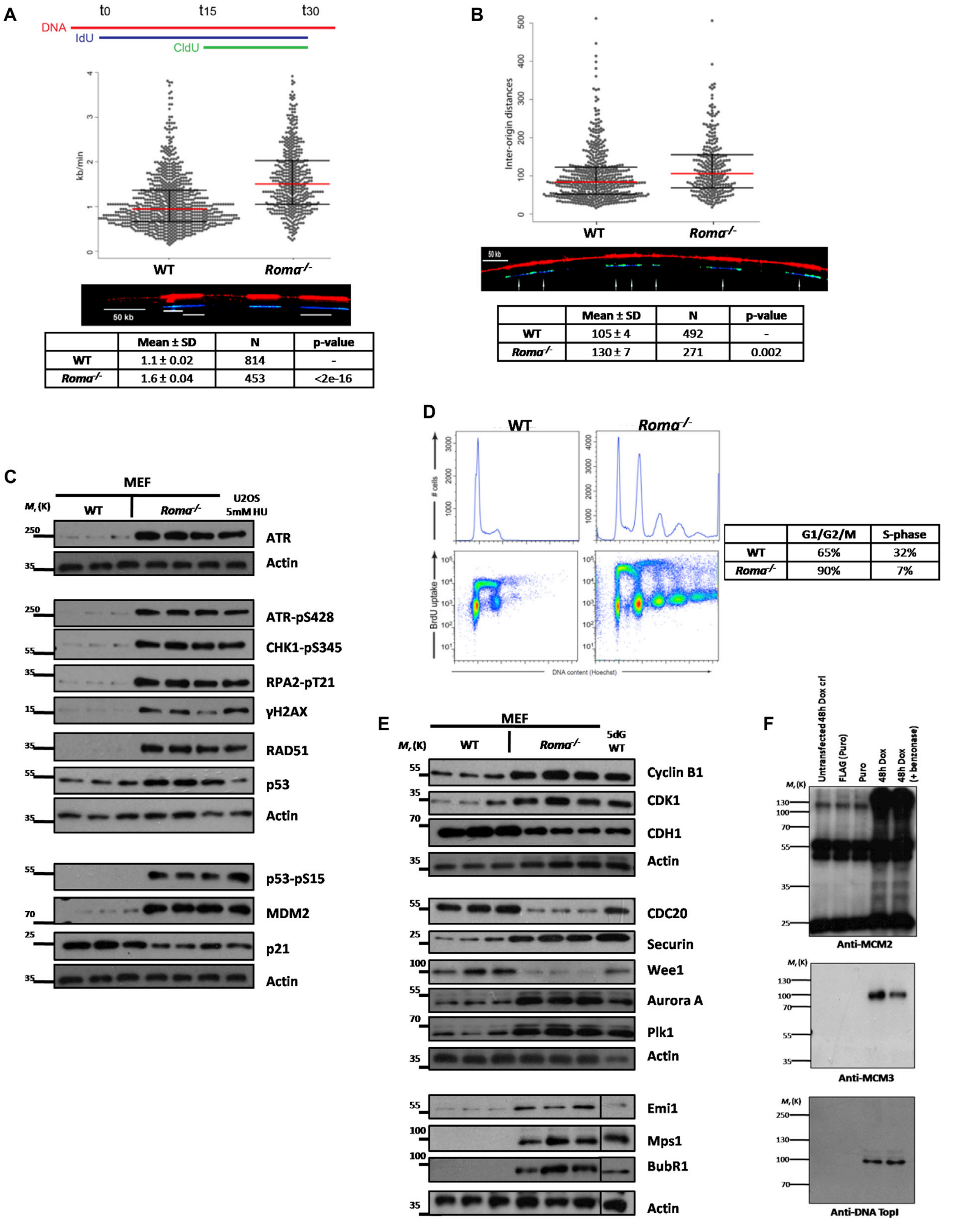


| | Mean ± SD | N | p-value |
|---|---|---|---|
| WT | 1.1 ± 0.02 | 814 | - |
| *Roma-/-* | 1.6 ± 0.04 | 453 | <2e-16 |

| | Mean ± SD | N | p-value |
|---|---|---|---|
| WT | 105 ± 4 | 492 | - |
| *Roma-/-* | 130 ± 7 | 271 | 0.002 |

| | G1/G2/M | S-phase |
|---|---|---|
| WT | 65% | 32% |
| *Roma-/-* | 90% | 7% |

*(legend on next page)*

checkpoint bypass resulting in polyploidy and genomic instability. This could predispose to tumorigenesis, and our previous study showed that Roma deficiency results in hyperplasia in mammary alveolar cells that have been depleted also of Gata3 (Oliver et al., 2012).

We show that the quiescence associated with differentiated MECs during lactation is dependent on appropriate levels of Roma. $p21^{Cip1}$ has long been implicated in terminal differentiation of multiple cell types: skeletal muscle myogenesis is associated with $p21^{Cip1}$ upregulation (Guo et al., 1995), and $p21^{Cip1}$-deficient keratinocytes exhibit reduced differentiation (Missero et al., 1996). Absence of $p21^{Cip1}$ has been implicated in enhancing appendage regeneration in mice through unscheduled S phase entry (Bedelbaeva et al., 2010). Likewise, in mammary gland development, Roma regulation of $p21^{Cip1}$ might be important in inducing quiescence in secretory epithelial cells during lactation. Furthermore, Roma is expressed at higher levels in basal epithelial cells, which cycle less frequently than luminal cells (Zeps et al., 1999).

Apart from preventing re-licensing and re-replication, geminin prevents over-cycling and exhaustion of multipotent progenitor populations and promotes genomic stability in long-term repopulating hematopoietic stem cells by inducing quiescence (Takihara, 2011). The transcriptional downregulation of geminin in the absence of Roma might be another contributing factor in the failure of *Roma*$^{-/-}$ MECs in lactating glands to enter G0. Interestingly, in human breast tissue, there is a lack of correlation between Mcm2–7 levels and markers of proliferation with one study showing that more than 50% of breast epithelial cells expressed Mcm2–7, although only 6% were proliferative (Stoeber et al., 2001). Thus, unlike other tissues, most breast epithelial cells are licensed but not actively cycling. This could render the mammary gland particularly sensitive to changes in the relative levels of cell-cycle regulators such as geminin and $p21^{Cip1}$. Hence, appropriate regulation of geminin by Roma could be essential as a safeguard against mammary tumorigenesis. We suggest that Roma and Stat6 interact in a negative transcriptional regulatory loop whereby Stat6 suppresses Roma expression specifically in luminal cells during pregnancy to allow elevated levels of proliferation while maintaining sufficient levels of Roma to prevent unscheduled DNA replication. Roma may have an additional role as an integral component of the replication fork, a notion that will require further investigation.

Although not described in mammary gland, physiological instances of re-replication and accumulation of polyploid cells do exist in the form of trophoblast giant (TG) cells and megakaryocytes, which have DNA contents between 8N to 64N. This results from endoreduplication in TG cells and endomitosis in megakaryocytes and has been associated with inhibition of CDK1 activity by p57, and suppression of DNA damage signaling by $p21^{Cip1}$ (Ullah et al., 2008). However, under non-physiological conditions, inactivation of $p21^{Cip1}$ can enhance endoreduplication, albeit leading to apoptosis (Jiang et al., 2000). The switch from mitotic cycles to endocycles has been linked to the degradation and loss of factors critical for mitotic entry such as cyclin B1, Plk1, and Aurora B by $APC^{Cdh1}$ (Edgar et al., 2014). However, given the upregulation of these factors in the absence of *Roma*, the accumulation of polyploid cells is likely to be a consequence of checkpoint escape and cell division failure.

We suggest, therefore, that Roma has a critical role in determining whether a cell can progress unperturbed through the cell division cycle and is required at precise stoichiometric levels in late G1/S phase to control replication fork progression. Roma appears also to be required in G2/M phase to ensure completion of cell division and genomic stability. The observation of hyperproliferation in gut, thymus, and spleen of *Roma*$^{-/-}$ mice implies a wider role for this transcriptional regulator. The identification of a critical role for Roma in controlling proliferation and genomic stability suggests that Roma may be a tumor suppressor, particularly in the breast, and awaits further studies.

## EXPERIMENTAL PROCEDURES

### Animals

Roma knockout mice were generated as described (Oliver et al., 2012). 7- to 8-week old virgin female mice were mated and plug-checked to confirm pregnancy. All animals were treated according to the local ethical committee (AWERB) and the UK Home Office guidelines.

### Tissue Sections and Immunofluorescence

Tissues were collected from abdominal glands and fixed in 4% paraformaldehyde overnight and stored in 70% ethanol. Tissues were embedded in wax and sectioned at 4-μm thickness. Sections were deparaffinized, and antigen was retrieved and stained as described in Oliver et al. (2012). Antibodies used were E-cadherin (BD Pharmingen, #610182), RPA32/RPA2 (pT21; Abcam, ab109394), H2AX (pS139; Millipore, 05-636), 53BP1 (Novus Biologicals, NB100-304), and DYKDDDDK Tag (Cell Signaling Technology, #2368). Slides were visualized with a Zeiss confocal microscope.

### PCR

RNA extraction of mammary tissue was carried out as described (Oliver et al., 2012). RT-PCR was performed using 2 μl of cDNA, and samples were cycled

**Figure 3. Altered Replication Dynamics and Aneuploidy in *Roma*-Deficient Cells**

(A) Primary MEFs were isolated from WT and *Roma*$^{-/-}$ embryos and cultured in vitro. DNA fibers from these cells were spread and stained with antibodies to IdU and CldU and counterstained with anti-DNA antibodies to ensure that broken fibers were not analyzed. Replication fork velocities were then measured. Data are presented as mean ± SD.

(B) Interorigin distances in WT and *Roma*$^{-/-}$ MEFs were measured. Data are presented as mean ± SD.

(C) Protein extracts were prepared from WT and *Roma*$^{-/-}$ primary MEFs followed by immunoblot analysis of markers of cell-cycle checkpoints and the DNA damage response. Extract from U2OS cells treated with 5 mM HU was used as a damage control.

(D) Primary WT and *Roma*$^{-/-}$ MEFs were pulsed with BrdU, and cell-cycle analysis was conducted. Percentage of cells in G1/G2/M and S phases was calculated.

(E) Immunoblot analysis of cell-cycle factors. Protein extract from 5dG WT gland was used as a proliferation control.

(F) EpH4 cells were transfected with a Roma-FLAG expression construct and cultured under puromycin selection. Doxycycline was used to induce Roma-FLAG expression. Protein extracts were prepared from EpH4 cells under the stated conditions followed by FLAG immunoprecipitation and immunoblot against Mcm2, Mcm3, and DNA Topoisomerase I showing association with Roma-FLAG.

See also Figure S3.

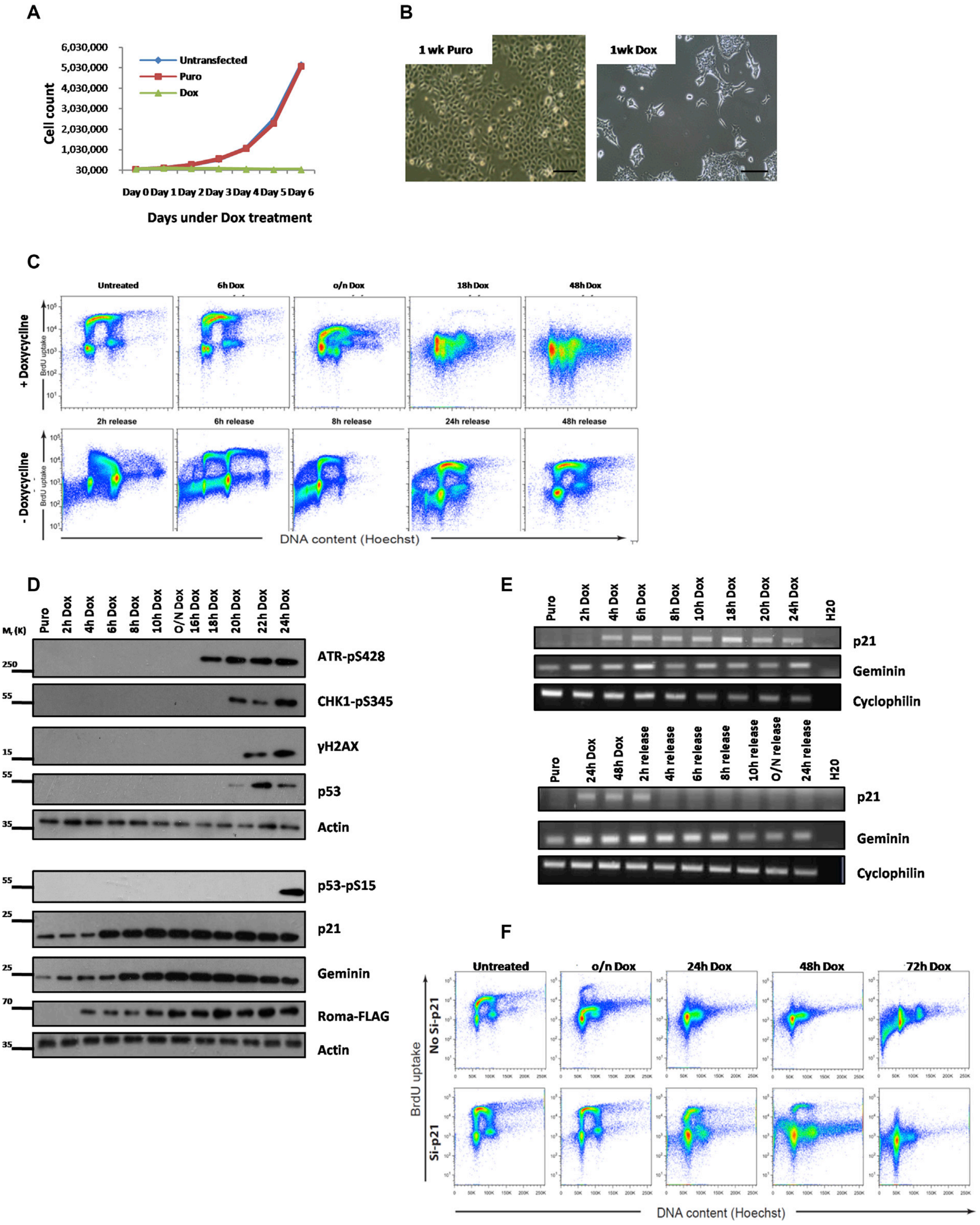


**Figure 4. Overexpression of Roma Results in S Phase Arrest**

(A) EpH4 cells were transfected with a Roma-FLAG expression construct and cultured under puromycin selection. Doxycycline was used to induce Roma-FLAG expression. Cell counts were monitored over the course of a week and represented in a graph.

(B) Representative images of cells under the indicated conditions.

(legend continued on next page)

27 times. Parameters were 95°C 5 min; 95°C 30 s, 60°C 30 s, 72°C 30 s; 72°C 7 min; 4°C hold. Primers used were p21$^{Cip1}$ forward (GCAGATCCACAGCGATATC); reverse (CAACTGCTCACTGTCCACG); Geminin forward (GGAGCATTGCTGTCTCTGAA); reverse (TCTTCAGCGTTCTCCTTGGG).

### Immunoblotting

Western blotting was performed as described (Oliver et al., 2012). Antibodies used were Cyclin D1 (Cell Signaling Technology, #2978), PCNA (Santa Cruz Biotechnology, sc-56), Geminin (Santa Cruz Biotechnology, FL-209), RPA32/RPA2 (pT21) (Abcam, ab109394), H2AX (pS139) (Millipore, 05-636), p53 (Santa Cruz Biotechnology, sc-126), p21 (BD Pharmingen, #556430), MDM2 (BD Pharmingen, #556353), DYKDDDDK Tag (Cell Signal, #2368), E2F1 (Bethyl, A300-766A), p53 (pS15) (Cell Signal, #9284), MDC1 (Bethyl, A300-053A), Cyclin E (Cell Signal, #4129), ATR (pS428) (Cell Signal, #2853), CHK1 (pS345) (Cell Signal, #2341), RAD51 (Abcam, ab63801), Cyclin B1 (Abcam, ab52187), Cdc6 (Santa Cruz Biotechnology, sc-8341), ORC3 (Santa Cruz Biotechnology, sc-23888), DNA polα (Santa Cruz Biotechnology, sc-365039), Cdk2 (Santa Cruz Biotechnology, sc-163), Cdk1 (Abcam, ab18), Wee1 (Abcam, ab137377), Rb (Santa Cruz Biotechnology, sc-50), Cdh1 (Abcam, ab154539), Cdc20 (Cell Signal, #4823), Securin (Abcam, ab3305), GINS (Santa Cruz Biotechnology, sc-373595), Cdc45 (Santa Cruz Biotechnology, sc-20685), Aurora A (Cell Signal, #3092), Plk1 (Abcam, ab17056), Gadd45 (Cell Signal, #4632), Emi1 (Santa Cruz Biotechnology, sc-30182), BubR1 (Santa Cruz Biotechnology, sc-16195), ATM (BD Pharmingen, 560007), ATM (pS1981) (Cell Signal, #4526), CHK2 (pT68) (Cell Signal, #16297), Cdt1 (Stavros Taraviras), and MCM3 (Nick Coleman).

### Primary Mammary Cell and Metaphase Preparation

Cells were isolated from mammary glands after a full natural wean according to Stingl et al. (2006) and cultured in complete media consisting of 10% fetal calf serum (FCS) in DMEM, gentamicin, 5 μg/ml insulin, and 10 ng/ml murine epidermal growth factor. Cells were treated with 0.1 μg/ml of nocodazole (Sigma-Aldrich). Cells were pelleted and 7ml of pre-warmed 0.05M KCl was added with gentle swirling followed by incubation for 12 min at 37°C. 10 ml of 3:1 methanol:acetic acid fixative was added dropwise before pelleting cells. R e-suspension in 5ml 3:1 methanol:acetic acid fixative was repeated thrice before cells were re- suspended in 2ml of 3:2 methanol:acetic acid fixative.

### Primary MEF Preparation

13.5-day-old embryos were harvested. Viscera were dissected away and carcasses were cut into small fragments. 3 ml trypsin/EDTA was added and incubated for 5 min at 37°C. Plates were rinsed with 5 ml 10% FCS in DMEM with gentamicin. Cells were re-suspended in 2 ml trypsin/EDTA and incubated for 5 min at 37°C. 8 ml of media was added and mixed by inversion before plating.

### In Vivo EdU Injections

500 μg of EdU (5-ethynyl-2′-deoxyuridine; Life Technologies) was administered at the various time points stated via intra-peritoneal injection 24 hr prior to tissue collection. Tissues were fixed in 4% paraformaldehyde for 2 hr at room temperature before transferring to 70% ethanol. Detection of EdU was done with the Click-iT EdU imaging kit (Invitrogen) as per manufacturer's instructions.

### Overexpression of Roma-FLAG in EpH4 Cells

Roma-FLAG was cloned into a PiggyBac transposon system, and EpH4 cells were transfected via the Amaxa nucleofector protocol (Lonza) as per manufacturer's instructions.

### Immunoprecipitation

Cells were harvested by scraping in cold PBS and re-suspended in RIPA lysis buffer with protease inhibitor cocktail. 2 mg of protein was used per immunoprecipitation. Anti-FLAG M2 affinity gel (Sigma-Aldrich, A2220) was prepared as per manufacturer's instructions and incubated with protein lysate overnight at 4°C with rotation. For immunoprecipitation with anti-Mcm2 (BD Transduction, 610700) and anti-DNA-TopI (Abcam, ab3825), Protein G sepharose 4 Fast Flow (GE Healthcare, GE17-0618-02) was used.

### Small Interfering RNA

ON-TARGETplus SMARTpool small interfering RNA (siRNA) against p21/Cdkn1a (Dharmacon, L-058636-00-0005) and ON-TARGETplus Non-targeting pool siRNA (Dharmacon, D-001810-10-05) as negative control were used.

### Cell-Cycle Analysis with BrdU

Cell-cycle analysis was done as described (Frey et al., 2014) with the minor modification that MEFs were trypsinized after the 30-min BrdU incorporation.

### Preparation, Spreading, and Immunolabeling of DNA Fibers

DNA fiber spreading was done as described (Frey et al., 2014) with minor modifications as follows: MEFs were incubated with 25 μM IdU for 15 min and supplied with fresh medium containing 25 μM CldU for another 15 min. Cells were trypsinized with 10× trypsin. Immunostaining and analysis were carried out as described (Guilbaud et al., 2011).

### Statistical Analysis

Statistical significance was assessed using unpaired two-tailed Student's t tests in Microsoft Excel (TTEST).

## SUPPLEMENTAL INFORMATION

Supplemental Information includes Supplemental Experimental Procedures, four figures, one table, and two movies and can be found with this article online at http://dx.doi.org/10.1016/j.celrep.2016.03.078.

## AUTHOR CONTRIBUTIONS

T.L.F.H. carried out the majority of the experiments except for the DNA fiber analyses, which were performed by G.G. T.L.F.H., G.G., J.J.B., J.E.S., and C.J.W. designed the work, analyzed the data, and wrote the manuscript.

## ACKNOWLEDGMENTS

We thank Helen Skelton for assistance with histology, Stavros Taraviras for the anti-Cdt1 antibody, and Nick Coleman for the anti-Mcm3 antibody. We thank Steve Jackson, Philip Zegerman, and Ashok Venkitaraman for helpful discussions. This work was supported by a PhD studentship from A*STAR Singapore to T.L.F.H. and funding from the Medical Research Council to C.J.W. G.G. and J.E.S. are supported by an MRC core grant to Laboratory of Molecular Biology (U105178808).

Received: July 29, 2015
Revised: December 18, 2015
Accepted: March 21, 2016
Published: April 14, 2016

(C) EpH4 cells transfected with a Roma-FLAG expression construct were treated with doxycycline at a series of time points and then released from doxycycline and pulsed with BrdU followed by flow cytometry analysis.
(D) Protein extracts were prepared from EpH4 cells at conditions stated and immunoblot analysis of cell-cycle checkpoint, and DNA damage response markers was conducted.
(E) RNA was extracted from EpH4 cells cultured in conditions stated followed by PCR analysis of p21Cip1.
(F) EpH4 cells cultured in conditions stated were treated with p21Cip1 siRNA and pulsed with BrdU followed by cell-cycle analysis using flow cytometry.
See also Figure S4.

## REFERENCES


Althoff, F., Karess, R.E., and Lehner, C.F. (2012). Spindle checkpoint-independent inhibition of mitotic chromosome segregation by Drosophila Mps1. Mol. Biol. Cell *23*, 2275–2291.

Asselin-Labat, M.L., Sutherland, K.D., Barker, H., Thomas, R., Shackleton, M., Forrest, N.C., Hartley, L., Robb, L., Grosveld, F.G., van der Wees, J., et al. (2007). Gata-3 is an essential regulator of mammary-gland morphogenesis and luminal-cell differentiation. Nat. Cell Biol. *9*, 201–209.

Bedelbaeva, K., Snyder, A., Gourevitch, D., Clark, L., Zhang, X.M., Leferovich, J., Cheverud, J.M., Lieberman, P., and Heber-Katz, E. (2010). Lack of p21 expression links cell cycle control and appendage regeneration in mice. Proc. Natl. Acad. Sci. USA *107*, 5845–5850.

Blow, J.J., and Ge, X.Q. (2008). Replication forks, chromatin loops and dormant replication origins. Genome Biol. *9*, 244.

Blow, J.J., and Hodgson, B. (2002). Replication licensing–defining the proliferative state? Trends Cell Biol. *12*, 72–78.

Burrell, R.A., McClelland, S.E., Endesfelder, D., Groth, P., Weller, M.C., Shaikh, N., Domingo, E., Kanu, N., Dewhurst, S.M., Gronroos, E., et al. (2013). Replication stress links structural and numerical cancer chromosomal instability. Nature *494*, 492–496.

Coqueret, O. (2003). New roles for p21 and p27 cell-cycle inhibitors: a function for each cell compartment? Trends Cell Biol. *13*, 65–70.

Edgar, B.A., Zielke, N., and Gutierrez, C. (2014). Endocycles: a recurrent evolutionary innovation for post-mitotic cell growth. Nat. Rev. Mol. Cell Biol. *15*, 197–210.

Faraldo, M.M., Deugnier, M.A., Tlouzeau, S., Thiery, J.P., and Glukhova, M.A. (2002). Perturbation of beta1-integrin function in involuting mammary gland results in premature dedifferentiation of secretory epithelial cells. Mol. Biol. Cell *13*, 3521–3531.

Floyd, S., Pines, J., and Lindon, C. (2008). APC/C Cdh1 targets aurora kinase to control reorganization of the mitotic spindle at anaphase. Curr. Biol. *18*, 1649–1658.

Frey, A., Listovsky, T., Guilbaud, G., Sarkies, P., and Sale, J.E. (2014). Histone H3.3 is required to maintain replication fork progression after UV damage. Curr. Biol. *24*, 2195–2201.

Guilbaud, G., Rappailles, A., Baker, A., Chen, C.L., Arneodo, A., Goldar, A., d'Aubenton-Carafa, Y., Thermes, C., Audit, B., and Hyrien, O. (2011). Evidence for sequential and increasing activation of replication origins along replication timing gradients in the human genome. PLoS Comput. Biol. *7*, e1002322.

Guo, K., Wang, J., Andrés, V., Smith, R.C., and Walsh, K. (1995). MyoD-induced expression of p21 inhibits cyclin-dependent kinase activity upon myocyte terminal differentiation. Mol. Cell. Biol. *15*, 3823–3829.

Harrigan, J.A., Belotserkovskaya, R., Coates, J., Dimitrova, D.S., Polo, S.E., Bradshaw, C.R., Fraser, P., and Jackson, S.P. (2011). Replication stress induces 53BP1-containing OPT domains in G1 cells. J. Cell Biol. *193*, 97–108.

Hosfield, D.J., Mol, C.D., Shen, B., and Tainer, J.A. (1998). Structure of the DNA repair and replication endonuclease and exonuclease FEN-1: coupling DNA and PCNA binding to FEN-1 activity. Cell *95*, 135–146.

Jiang, Z., Liang, P., Leng, R., Guo, Z., Liu, Y., Liu, X., Bubnic, S., Keating, A., Murray, D., Goss, P., and Zacksenhaus, E. (2000). E2F1 and p53 are dispensable, whereas p21(Waf1/Cip1) cooperates with Rb to restrict endoreduplication and apoptosis during skeletal myogenesis. Dev. Biol. *227*, 8–41.

Khaled, W.T., Read, E.K., Nicholson, S.E., Baxter, F.O., Brennan, A.J., Came, P.J., Sprigg, N., McKenzie, A.N., and Watson, C.J. (2007). The IL-4/IL-13/Stat6 signalling pathway promotes luminal mammary epithelial cell development. Development *134*, 2739–2750.

Khan, S.H., and Wahl, G.M. (1998). p53 and pRb prevent rereplication in response to microtubule inhibitors by mediating a reversible G1 arrest. Cancer Res. *58*, 396–401.

Klotz-Noack, K., McIntosh, D., Schurch, N., Pratt, N., and Blow, J.J. (2012). Re-replication induced by geminin depletion occurs from G2 and is enhanced by checkpoint activation. J. Cell Sci. *125*, 2436–2445.

Kouros-Mehr, H., Slorach, E.M., Sternlicht, M.D., and Werb, Z. (2006). GATA-3 maintains the differentiation of the luminal cell fate in the mammary gland. Cell *127*, 1041–1055.

Lianga, N., Williams, E.C., Kennedy, E.K., Doré, C., Pilon, S., Girard, S.L., Deneault, J.S., and Rudner, A.D. (2013). A Wee1 checkpoint inhibits anaphase onset. J. Cell Biol. *201*, 843–862.

Lukas, C., Savic, V., Bekker-Jensen, S., Doil, C., Neumann, B., Pedersen, R.S., Grøfte, M., Chan, K.L., Hickson, I.D., Bartek, J., and Lukas, J. (2011). 53BP1 nuclear bodies form around DNA lesions generated by mitotic transmission of chromosomes under replication stress. Nat. Cell Biol. *13*, 243–253.

Lupo, A., Cesaro, E., Montano, G., Zurlo, D., Izzo, P., and Costanzo, P. (2013). KRAB-Zinc Finger Proteins: A Repressor Family Displaying Multiple Biological Functions. Curr. Genomics *14*, 268–278.

Mazouzi, A., Velimezi, G., and Loizou, J.I. (2014). DNA replication stress: causes, resolution and disease. Exp. Cell Res. *329*, 85–93.

Melixetian, M., Ballabeni, A., Masiero, L., Gasparini, P., Zamponi, R., Bartek, J., Lukas, J., and Helin, K. (2004). Loss of Geminin induces rereplication in the presence of functional p53. J. Cell Biol. *165*, 473–482.

Missero, C., Di Cunto, F., Kiyokawa, H., Koff, A., and Dotto, G.P. (1996). The absence of p21Cip1/WAF1 alters keratinocyte growth and differentiation and promotes ras-tumor progression. Genes Dev. *10*, 3065–3075.

Myung, K., Datta, A., and Kolodner, R.D. (2001). Suppression of spontaneous chromosomal rearrangements by S phase checkpoint functions in Saccharomyces cerevisiae. Cell *104*, 397–408.

Niculescu, A.B., 3rd, Chen, X., Smeets, M., Hengst, L., Prives, C., and Reed, S.I. (1998). Effects of p21(Cip1/Waf1) at both the G1/S and the G2/M cell cycle transitions: pRb is a critical determinant in blocking DNA replication and in preventing endoreduplication. Mol. Cell. Biol. *18*, 629–643.

Oliver, C.H., Khaled, W.T., Frend, H., Nichols, J., and Watson, C.J. (2012). The Stat6-regulated KRAB domain zinc finger protein Zfp157 regulates the balance of lineages in mammary glands and compensates for loss of Gata-3. Genes Dev. *26*, 1086–1097.

Petronczki, M., Lénárt, P., and Peters, J.M. (2008). Polo on the Rise-from Mitotic Entry to Cytokinesis with Plk1. Dev. Cell *14*, 646–659.

Sexton, D.J., Berdis, A.J., and Benkovic, S.J. (1997). Assembly and disassembly of DNA polymerase holoenzyme. Curr. Opin. Chem. Biol. *1*, 316–322.

Shaltiel, I.A., Krenning, L., Bruinsma, W., and Medema, R.H. (2015). The same, only different - DNA damage checkpoints and their reversal throughout the cell cycle. J. Cell Sci. *128*, 607–620.

Stewart, Z.A., Leach, S.D., and Pietenpol, J.A. (1999). p21(Waf1/Cip1) inhibition of cyclin E/Cdk2 activity prevents endoreduplication after mitotic spindle disruption. Mol. Cell. Biol. *19*, 205–215.

Stingl, J., Eirew, P., Ricketson, I., Shackleton, M., Vaillant, F., Choi, D., Li, H.I., and Eaves, C.J. (2006). Purification and unique properties of mammary epithelial stem cells. Nature *439*, 993–997.

Stoeber, K., Tlsty, T.D., Happerfield, L., Thomas, G.A., Romanov, S., Bobrow, L., Williams, E.D., and Williams, G.H. (2001). DNA replication licensing and human cell proliferation. J. Cell Sci. *114*, 2027–2041.

Takihara, Y. (2011). Role for Geminin in sustaining the activity of hematopoietic stem cells. Cell Cycle *10*, 561–562.

Toledo, L.I., Altmeyer, M., Rask, M.B., Lukas, C., Larsen, D.H., Povlsen, L.K., Bekker-Jensen, S., Mailand, N., Bartek, J., and Lukas, J. (2013). ATR prohibits replication catastrophe by preventing global exhaustion of RPA. Cell *155*, 1088–1103.

Ullah, Z., Kohn, M.J., Yagi, R., Vassilev, L.T., and DePamphilis, M.L. (2008). Differentiation of trophoblast stem cells into giant cells is triggered by p57/Kip2 inhibition of CDK1 activity. Genes Dev. *22*, 3024–3036.

Urrutia, R. (2003). KRAB-containing zinc-finger repressor proteins. Genome Biol. *4*, 231.

Waga, S., Hannon, G.J., Beach, D., and Stillman, B. (1994). The p21 inhibitor of cyclin-dependent kinases controls DNA replication by interaction with PCNA. Nature *369*, 574–578.

Watson, C.J., and Khaled, W.T. (2008). Mammary development in the embryo and adult: a journey of morphogenesis and commitment. Development *135*, 995–1003.

Zeps, N., Bentel, J.M., Papadimitriou, J.M., and Dawkins, H.J. (1999). Murine progesterone receptor expression in proliferating mammary epithelial cells during normal pubertal development and adult estrous cycle. Association with eralpha and erbeta status. J. Histochem. Cytochem. *47*, 1323–1330.

Cell Reports, Volume *15*

## Supplemental Information

# The KRAB Zinc Finger Protein Roma/Zfp157 Is a Critical Regulator of Cell-Cycle Progression and Genomic Stability

**Teresa L.F. Ho, Guillaume Guilbaud, J. Julian Blow, Julian E. Sale, and Christine J. Watson**

# Figure S1 (related to Figure 1)

## A

| 5dG | | | | | | |
|---|---|---|---|---|---|---|
| | **WT1** | **WT2** | **WT3** | ***Roma$^{-/-}$*1** | ***Roma$^{-/-}$*2** | ***Roma$^{-/-}$*3** |
| **AVG%** | 21.69 | 25.46 | 24.94 | 46.01 | 45.96 | 47.31 |
| **SD** | 8.35 | 8.73 | 9.16 | 6.45 | 5.79 | 6.02 |
| p = 9.52E-04 | | | | | | |
| **10dL** | | | | | | |
| | **WT1** | **WT2** | **WT3** | ***Roma$^{-/-}$*1** | ***Roma$^{-/-}$*2** | ***Roma$^{-/-}$*3** |
| **AVG%** | 4.04 | 4.95 | 4.78 | 29.02 | 27.8 | 29.5 |
| **SD** | 6.58 | 5.43 | 6.95 | 12.48 | 9.14 | 10.46 |
| p = 2.49E-05 | | | | | | |

## B

| Small Intestine | | | | | | |
|---|---|---|---|---|---|---|
| | **WT1** | **WT2** | **WT3** | ***Roma$^{-/-}$*1** | ***Roma$^{-/-}$*2** | ***Roma$^{-/-}$*3** |
| **AVG%** | 29.35 | 29.92 | 30.10 | 45.47 | 44.58 | 44.97 |
| **SD** | 1.65 | 0.34 | 0.57 | 1.13 | 0.62 | 0.30 |
| p = 1.84E-06 | | | | | | |
| | | | | | | |
| **Spleen** | | | | | | |
| | **WT1** | **WT2** | **WT3** | ***Roma$^{-/-}$*1** | ***Roma$^{-/-}$*2** | ***Roma$^{-/-}$*3** |
| **AVG%** | 2.25 | 1.73 | 1.78 | 8.98 | 9.27 | 8.89 |
| **SD** | 0.49 | 0.19 | 0.18 | 0.41 | 0.80 | 0.43 |
| p = 1.19E-05 | | | | | | |
| | | | | | | |
| **Thymus** | | | | | | |
| | **WT1** | **WT2** | **WT3** | ***Roma$^{-/-}$*1** | ***Roma$^{-/-}$*2** | ***Roma$^{-/-}$*3** |
| **AVG%** | 3.39 | 3.16 | 3.36 | 20.11 | 20.01 | 19.75 |
| **SD** | 0.29 | 0.39 | 0.25 | 0.57 | 0.13 | 0.15 |
| p = 1.65E-07 | | | | | | |

## C

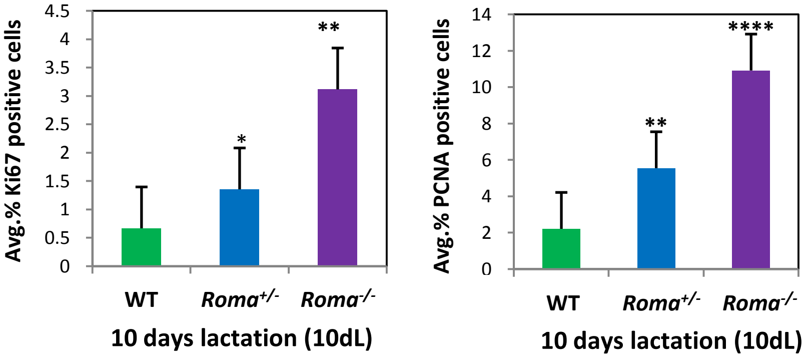


## D

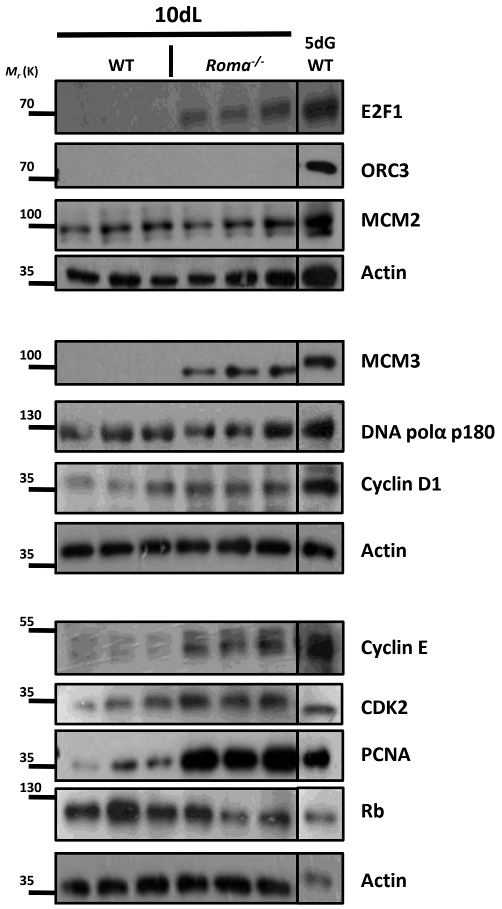

# Figure S2 (Related to Figure 2)

A

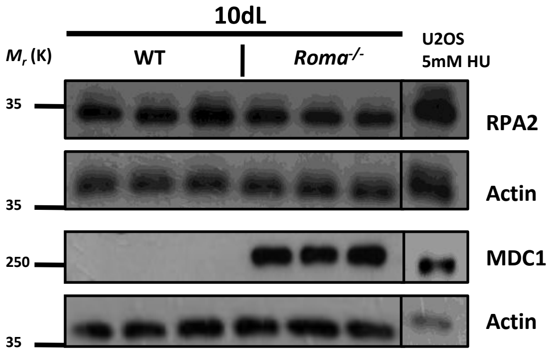


B

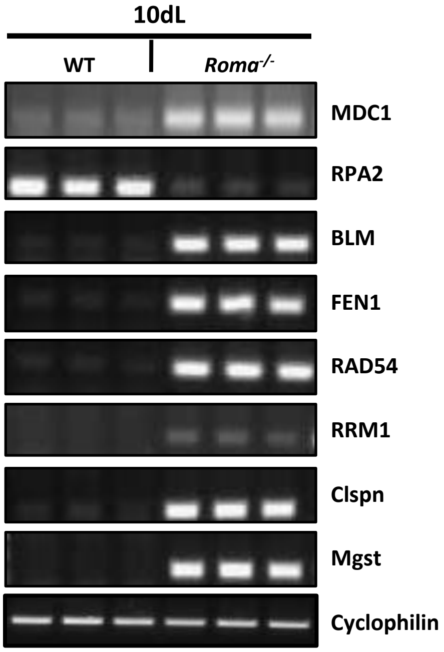


C

| WT | Chr 1 | Chr 2 | Chr 3 |
|---|---|---|---|
| Breaks | | 1 | |
| Fragile site | 2 | | 2 |
| Deletions | | | |
| Translocations | | | |
| Duplication | | | |

| *Roma*-/- | Chr1 | Chr2 | Chr4 | Chr5 | Chr6 | Chr7 | Chr8 | Chr9 | Chr10 | Chr11 | Chr12 | Chr14 | Chr15 | Chr16 | Chr17 | Chr18 | ChrX |
|---|---|---|---|---|---|---|---|---|---|---|---|---|---|---|---|---|---|
| Breaks | 6 | 5 | 3 | 9 | 6 | 2 | 3 | 3 | | 4 | 2 | 1 | 2 | 7 | 3 | 2 | 2 |
| Fragile site | 5 | 3 | 7 | 3 | 3 | 2 | | 2 | 10 | 2 | | | | 3 | | 1 | 2 |
| Deletions | 4 | 4 | 8 | 6 | 2 | 2 | 2 | 1 | | 2 | 2 | 4 | 2 | 3 | | 3 | 1 |
| Translocations | | 2&14 | 4&12<br>4&18 | | 6&2<br>6&17 | 7&12<br>7&17 | | 9&18 | | 11&12<br>11&13<br>11&15 | | 14&3<br>14&15<br>14&17 | | 16&19 | 17&3<br>17&8<br>17&9<br>17&19 | 18&12 | X&3<br>X&10<br>X&13 |
| Duplication | 2 | | 3 | 3 | | 2 | | 2 | | 1 | 1 | 2 | | | | | |

| *Roma*-/- | Chr1 | Chr2 | Chr5 | Chr6 | Chr7 | Chr8 | Chr10 | Chr11 | Chr12 | Chr13 | Chr14 | Chr15 | Chr16 | Chr17 | Chr18 | Chr19 | ChrX |
|---|---|---|---|---|---|---|---|---|---|---|---|---|---|---|---|---|---|
| Premature separation of sister chromatids (freq.) | 1 | 4 | 2 | 2 | 2 | 4 | 15 | 10 | 1 | 5 | 4 | 6 | 1 | 5 | 4 | 1 | 3 |

# Figure S3 (Related to Figure 3)

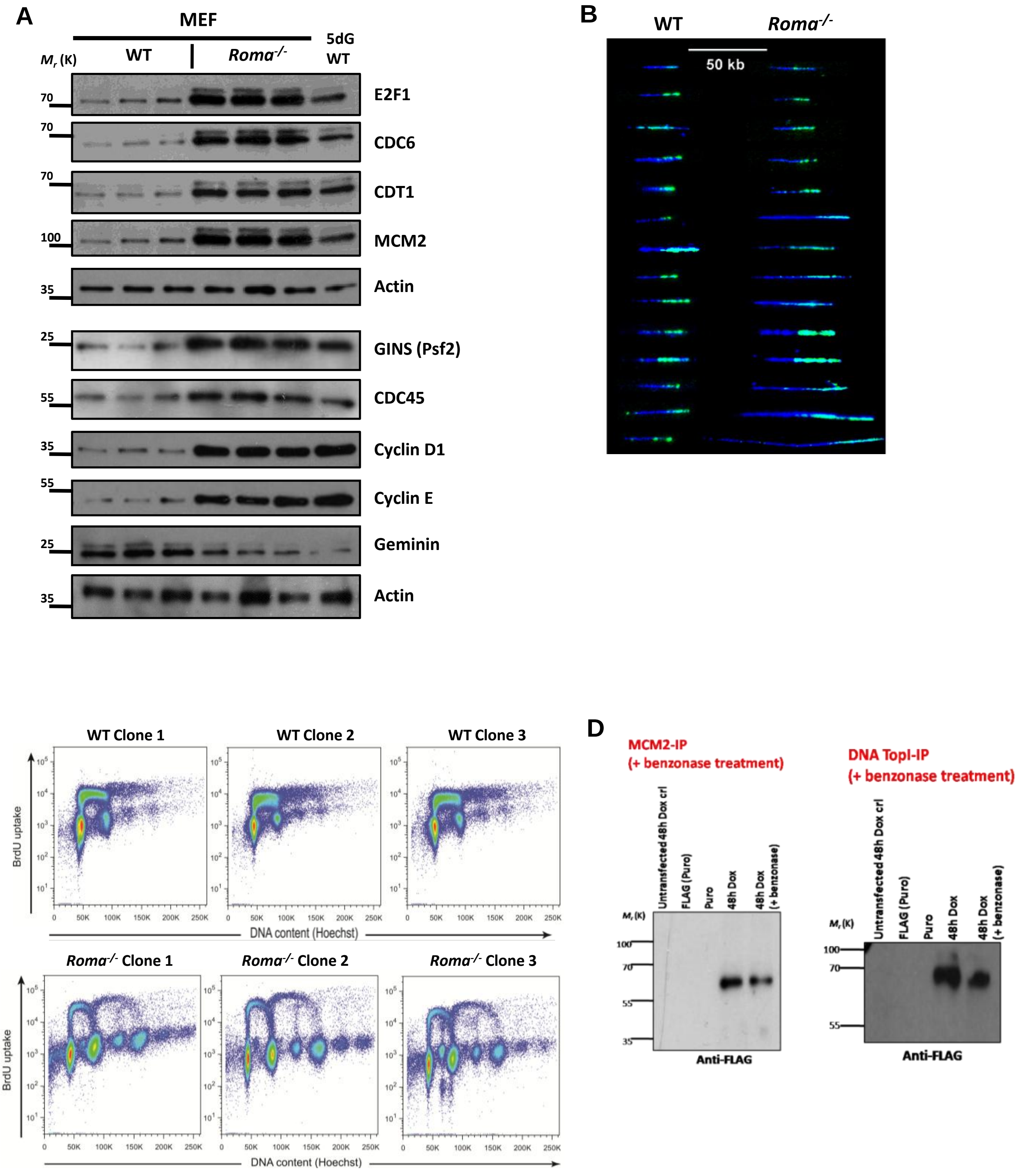

# Figure S4 (Related to Figure 4)

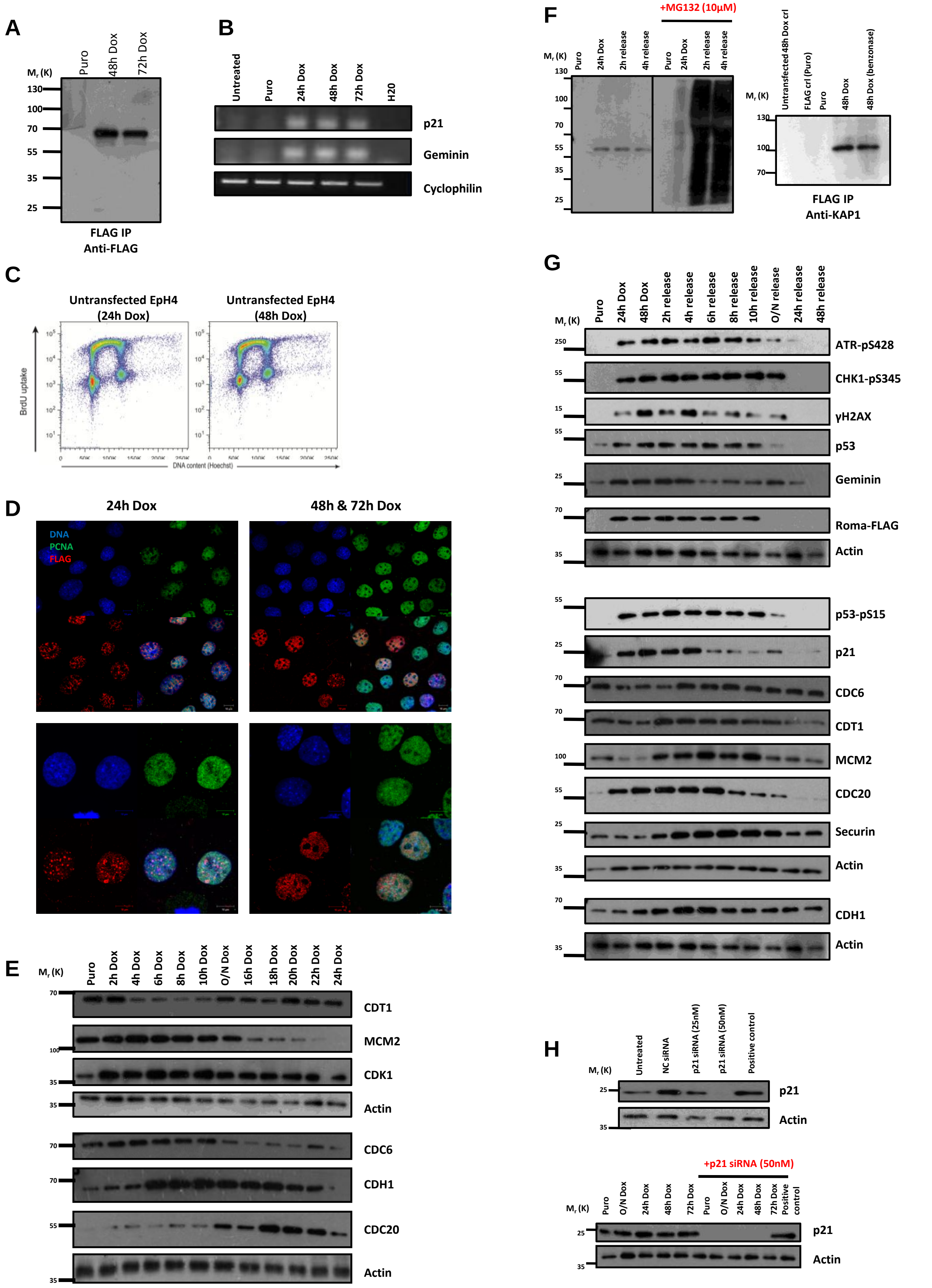

## Supplemental Figure Legends

**Figure S1 (Related to Figure 1) (A)** Average percentages of EdU+ cells per alveolar structure in WT and *Roma*$^{-/-}$ mammary glands at 5dG and 10dL (n=3 mice per group, 5dG: p = 9.52E-04, 10dL: p = 2.49E-05, Student's t-test). **(B)** *In vivo* IP injections of EdU in WT and *Roma*$^{-/-}$ mice at 7-8 weeks of age and various organs harvested. Number of EdU+ cells per field of view were counted at 20X magnification for small intestine and 40X magnification for spleen and thymus and presented as an average percentage of positive cells to total cells counted. (n=3 mice per group, Small intestine: p = 1.84E-06, Spleen: p = 1.19E-05, Thymus: p = 1.65E-07, Student's t-test). **(C)** Immunofluorescence analysis of WT, *Roma*$^{+/-}$ and *Roma*$^{-/-}$ mammary glands at 10dL with Ki67 and PCNA. Average percentages of Ki67+ or PCNA+ cells per field of view at 20X magnification were calculated. Data is presented as mean and error bars represent standard deviation (SD). (n=3 mice per group, Ki67: WT vs *Roma*$^{+/-}$: p = 0.024; WT vs *Roma*$^{-/-}$: p = 0.008; PCNA: WT vs *Roma*$^{+/-}$: p = 0.001; WT vs *Roma*$^{-/-}$: p = 4.73E-06; Student's t-test). **(D)** Protein extracts were prepared from WT and *Roma*$^{-/-}$ mammary glands at 10dL (n=3 mice per group) followed by immunoblot analysis of cell cycle factors. Extract from 5dG WT gland used as proliferation control.

**Figure S2 (Related to Figure 2) (A)** Immunoblot analysis of cell cycle checkpoints and DNA damage response markers in WT and *Roma*$^{-/-}$ mammary glands at 10dL (n=3 mice per group). Extract from U2OS cells treated with 5 mM HU was used as a damage control**. (B)** PCR analysis of various cell cycle checkpoints and DNA damage response markers in WT and *Roma*$^{-/-}$ mammary glands at 10dL (n=3 mice per group) were conducted. Cyclophilin used as an internal control. **(C)** Summary of gross chromosomal rearrangements in WT and *Roma*$^{-/-}$ mammary epithelial cells isolated from mammary glands after a full natural wean (n=3 mice per group).

**Figure S3 (Related to Figure 3) (A)** Immunoblot analysis of cell cycle factors in WT and *Roma*$^{-/-}$ MEFs (n=3 clones per group). Extract from 5dG WT gland was used as a proliferation control. **(B)** A selection of representative fibers analysed. **(C)** Primary WT and *Roma*$^{-/-}$ MEFs at passage 5 were pulsed with BrdU followed by cell cycle analysis using flow cytometry (n=3 clones per group). **(D)** Protein extracts were prepared from EpH4 cells under the following conditions: untransfected EpH4 cells+48h doxycycline, EpH4+control FLAG vector (Puromycin selection), EpH4+Roma-FLAG vector (Puromycin selection, no induction), EpH4+Roma-FLAG vector + 48h doxycycline (Puromycin selection, induction), EpH4+Roma-FLAG vector+48h doxycycline+benzonase treatment (Puromycin selection, induction) followed by Mcm2 and DNA Topoisomerase I immunoprecipitation respectively and immunoblot against Roma-FLAG showing association.

**Figure S4 (Related to Figure 4) (A)** Protein extracts were prepared from EpH4 cells under puromycin selection (without doxycycline induction) and at various timepoints after doxycycline-induced Roma-FLAG expression. FLAG immunoprecipitation was conducted followed by immunoblot analysis of Roma-FLAG expression. **(B)** RNA was extracted from EpH4 cells under puromycin selection (without doxycycline induction) and at various timepoints after doxycycline-induced Roma-FLAG expression and PCR analysis of p21$^{Cip1}$ and geminin was conducted. Cyclophilin used as an internal control. **(C)** Untransfected EpH4 cells were treated with doxycyline for 48h before pulsing with BrdU followed by cell cycle analysis. **(D)** Representative immunofluorescence analysis of PCNA and Roma-FLAG in EpH4 cells at various timepoints after doxycycline-induced Roma-FLAG expression. Scale bars = 10µm. **(E)** Immunoblot analysis of cell cycle factors in EpH4 cells during a timecourse of doxycycline-induced Roma-FLAG

expression was conducted**.** **(F)** Protein was extracted from EpH4 cells over a timecourse of release from doxycycline-induced Roma-FLAG expression (+/- MG132) followed by anti-FLAG immunoblot analysis. Protein extracts were prepared from EpH4 cells under the stated conditions followed by FLAG immunoprecipitation and immunoblot against KAP1 showing association with Roma-FLAG. **(G)** Immunoblot analysis of cycle checkpoint, DNA damage response and cell cycle factors in EpH4 cells during a timecourse of release from doxycycline-induced Roma-FLAG expression was conducted**. (H)** Protein was extracted from EpH4 cells under the following treatment conditions – untreated, scrambled siRNA, 25nM $p21^{Cip1}$ siRNA, 50nM $p21^{Cip1}$ siRNA followed by immunoblot for $p21^{Cip1}$. Extract from 5dG WT gland was used as a proliferation control. Protein was extracted from EpH4 cells under the stated treatment conditions (+/- 50nM $p21^{Cip1}$ siRNA) followed by immunoblot against $p21^{Cip1}$.

**Video S1 (Related to Figure 3)**

Video clip of WT MEFs during live cell imaging.

**Video S2 (Related to Figure 3)**

Video clip of *Roma*$^{-/-}$ MEFs during live cell imaging.

**Table S1 (Related to Figure 3)**

A separate Excel Workbook containing raw data from DNA fiber analysis.

## Supplemental Experimental Procedures

### Quantification of EdU-positive cells in other tissues

For intestine, images were taken at 20X magnification and 10 fields of view were counted. For spleen and thymus, images were taken at 40X magnification and 5 fields of view were counted. Analysis was conducted as stated in Experimental Procedures.

### Quantification of PCNA and Ki67-positive MECs

For the analysis of PCNA+ and Ki67+ cells, images were taken at 20X magnification and 10 fields of view were analyzed per gland. Number of PCNA+ or Ki67+ cells were expressed as a percentage of total number of cells. Statistical significance was assessed using unpaired two-tailed Student's *t*-tests in Microsoft Excel (TTEST).

### PCR

Other primers used include (PCR parameters can be found under Experimental Procedures):

Cyclin B1 – forward (CAGAGTTCTGAACTTCAGCCTG); reverse (TTGTGAGGCCACAGTTCACCAT)

CDK1 – forward (ACAGAGAGGGTCCGTCGTAA); reverse (ATTGCAGTACTGGGCACTCC)

MDM2 – forward (TTAGTGGCTGTAAGTCAGCAAGA); reverse (CCTTCAGATCACTCCCACCT)

RPA2 – forward (TGTTGGCGGCAGAATCATGG; reverse (CTCGTCAAGTGGCTCCATCAA)

BLM – forward (AAGCCTGAGTGAGGATCATGG); reverse (TGACAGACACATCACCCTCTG)

FEN1 – forward (GCTAGCTGCTTAAGGCTCGT); reverse (AGGAGCAATGGCTTCTTCCTAC)

RAD54 – forward (TGGACCCAAGCCTCATCCTC)

reverse (TAAGCTCCTCCTCATCCTGGC)

RRM1 – forward (ACGAAGCACCCTGACTATGC); reverse (TGGCAGAATTCAGGCGATCC)

Clspn – forward (GCACTGCAGAAGAATGCCAG); reverse (TTCCTTGAGTTTCGGGGAGC)

Mgst – forward (AAGATTGGAAGCATGGCCGA); reverse (CTGGGTTGGCAAAAACCTTGT)
